# Structural and biochemical characterization of Rv0187, an O-methyltransferase from *Mycobacterium tuberculosis*

**DOI:** 10.1038/s41598-019-44592-7

**Published:** 2019-05-30

**Authors:** Sanghyun Lee, Jihoon Kang, Jungwook Kim

**Affiliations:** Department of Chemistry, Gwangju Institute of Science and Technology, Gwangju, 61005 Republic of Korea

**Keywords:** Transferases, X-ray crystallography

## Abstract

Catechol O-methyltransferase (COMT) is widely distributed in nature and installs a methyl group onto one of the vicinal hydroxyl groups of a catechol derivative. Enzymes belonging to this family require two cofactors for methyl transfer: S-adenosyl-l-methionine as a methyl donor and a divalent metal cation for regiospecific binding and activation of a substrate. We have determined two high-resolution crystal structures of Rv0187, one of three COMT paralogs from *Mycobacterium tuberculosis*, in the presence and absence of cofactors. The cofactor-bound structure clearly locates strontium ions and S-adenosyl-l-homocysteine in the active site, and together with the complementary structure of the ligand-free form, it suggests conformational dynamics induced by the binding of cofactors. Examination of *in vitro* activities revealed promiscuous substrate specificity and relaxed regioselectivity against various catechol-like compounds. Unexpectedly, mutation of the proposed catalytic lysine residue did not abolish activity but altered the overall landscape of regiospecific methylation.

## Introduction

**Figure 1:**
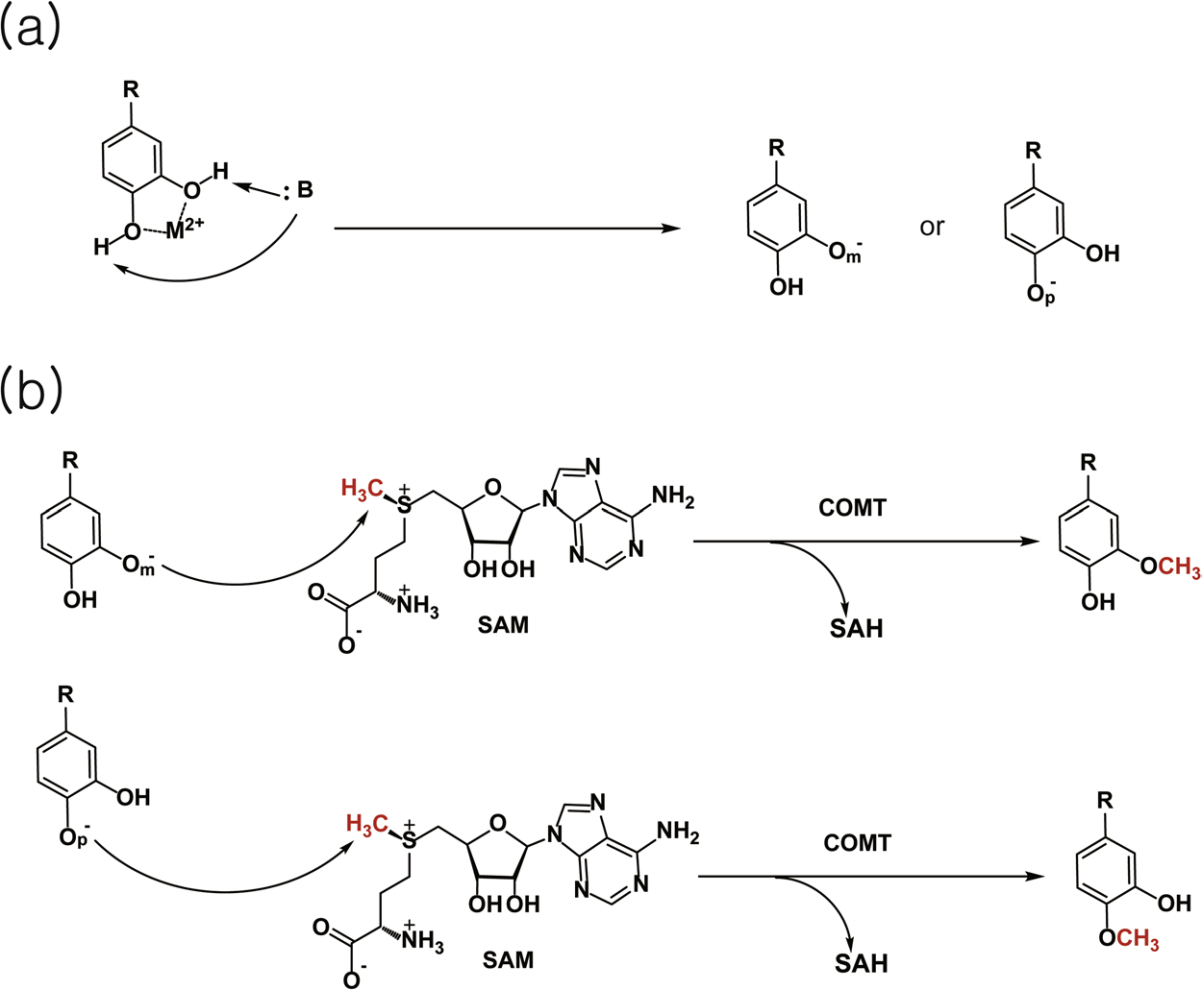
Overall reaction scheme for catechol O-methyltransferase. COMT-dependent methyltransfer initiates with (**a**) regioselective deprotonation of a hydroxyl group on a catechol-like substrate. (**b**) In the subsequent step, the activated hydroxyl group attacks the electrophilic methyl group of SAM in an SN2-manner, yielding a methylated catechol-like product at the meta (top) or para (bottom) position. M^2+^ and :B denote a divalent metal ion and a general base, respectively.

Catechol O-methyltransferase (COMT) is widely conserved in bacteria, plants, fungi, and metazoa. The chemical reaction catalyzed by this family (pfam methyltransf_3, or PF01596) involves the transfer of a methyl group from S-adenosyl-l-methionine (SAM) to a hydroxyl group of catechol-like compounds (Fig. 1). These enzymes are class I O-methyltransferases (OMTs) and typically composed of 200–250 amino acids and require a divalent metal ion for full activity. Class II OMTs, on the other hand, are relatively larger and metal independent. The biological function of mammalian COMTs is best characterized in the metabolism of catecholamines, including dopamine, norepinephrine, and epinephrine. Most metal-dependent plant COMTs strictly target caffeoyl-CoA, a key intermediate in lignin biosynthesis. Other cation-dependent OMTs in eukaryotes display promiscuous substrate specificity; examples include phenylpropanoid and flavonoid O-methyltransferase (PFOMT) from the ice plant *Mesembryanthemum crystallinum* acting on phenylpropanoid and flavonoid conjugates^1^ and *Podospora anserina* O-methyltransferase (PaOMT or PaMTH1), which methylates flavonoids containing vicinal hydroxyl groups such as myricetin or quercetin^2–4^. Cellular targets of bacterial COMTs appear to be more diverse and often unknown, in contrast to the targets of their eukaryotic homologs. Physiological roles of bacterial enzymes have been linked to the biosynthesis of antibiotics in several soil bacteria^5–10^, posttranslational modification of an outer membrane protein in *Anaplasma phagocytophilum*^11^, and posttranscriptional modification of tRNA in *Bacillus subtilis*^12^.

X-ray crystal structures of eukaryotic COMTs from rat (RnCOMT), human (hCOMT), *Sorghum bicolor* (SbCCoAOMT), and *Medicago sativa* (MsCCoAOMT) revealed the presence of a divalent cation (*i.e*., magnesium or calcium ion) in the active site, coordinated by two aspartates and one asparagine residue, which are essentially invariant within this family (Supplementary Fig. 1)^13–16^. Twenty crystal structures of bacterial COMTs have been reported from 14 species to date, and eight of them contain a divalent cation in the conserved metal-binding site, *e.g*., Mg^2+^, Mn^2+^, and Ni^2+^ ^11,12,17–19^. The divalent ion plays an important role in productive binding of the catechol moiety of a substrate and has been suggested to lower the pKa of a nucleophilic hydroxyl group to facilitate methyl transfer^13^. The correct orientation of vicinal hydroxyl groups for chelating the metal ion leads to regiospecific methylation by COMTs, which can be attributed to the relative nucleophilicity between two hydroxyl groups and the hydrophobicity of the substituent (-R in Fig. 1) of a catechol substrate. While eukaryotic COMTs predominantly methylate the meta position of their biological targets, the regioselectivity of bacterial homologs is less clear largely because most of their *in vivo* substrates are unknown.

*Mycobacterium tuberculosis* (MTB) H37Rv is predicted to encode three COMTs, *i.e*., Rv1220, Rv1703, and Rv0187, which share 29 to 36% amino acid sequence identity. Detailed cellular functions of these paralogs are still elusive. Among these COMTs, the crystal structure of Rv1220 has been determined, which surprisingly displays a lack of the signature metal-binding site conserved in Class I COMTs (Supplementary Fig. 1)^20^. Recently, a crystal structure of TomG, a homolog of MTB Rv0187 sharing 54% sequence identity, has been reported along with its biological role in the assembly of an antitumor antibiotic agent, tomaymycin^9,21^. The authors demonstrated that the enzyme is able to install a methyl group on the 5-hydroxyl group of 4,5-dihydroxyanthranilic acid both *in vivo* and *in vitro*. Meanwhile, whether MTB encodes analogous enzymes to synthesize tomaymycin has not been established. Furthermore, neither the structural nor biochemical properties of Rv0187 have been reported to date; thus, it is exceedingly challenging to predict its biological function beyond annotation transfer by homology.

Here, we present two crystal structures of Rv0187 from MTB H37Rv with and without cofactors at 1.64 and 2.08 Å resolution, respectively. These structures provide snapshots of the conformational dynamics of the protein upon the binding of cofactors, *i.e*., divalent metal ions and methyl donors. In addition, the substrate specificity, regioselectivity, and metal dependency of the enzyme have been examined using a panel of compounds bearing the catechol moiety for *in vitro* methyl transfer activity. Combined with genetic analysis results, our structural and biochemical data provide valuable insights into the biological function of Rv0187.

## Results

### Solution properties of recombinant Rv0187

We tested several expression constructs of Rv0187 to facilitate purification and crystallization of the recombinant protein. Notably, size-exclusion chromatography (SEC) of the purified protein sample exhibits slightly different profiles among constructs. When the protein construct of Rv0187 fused with a C-terminal His6-tag was analyzed, a minor peak appeared close to the void volume of the column, and a major peak emerged around the elution volume consistent with that of a dimeric form, which is the typical oligomeric state of class I COMTs (Supplementary Fig. 2). The ratio between high oligomer and the dimer is reversed in the case of an N-terminal His6-tagged sample, where the protein elutes as high-order oligomers more predominantly than in the C-terminal His6-tagged version. Fractions corresponding to dimers were pooled and used for further biochemical and structural studies.

### Overall structure of Rv0187

**Figure 2:**
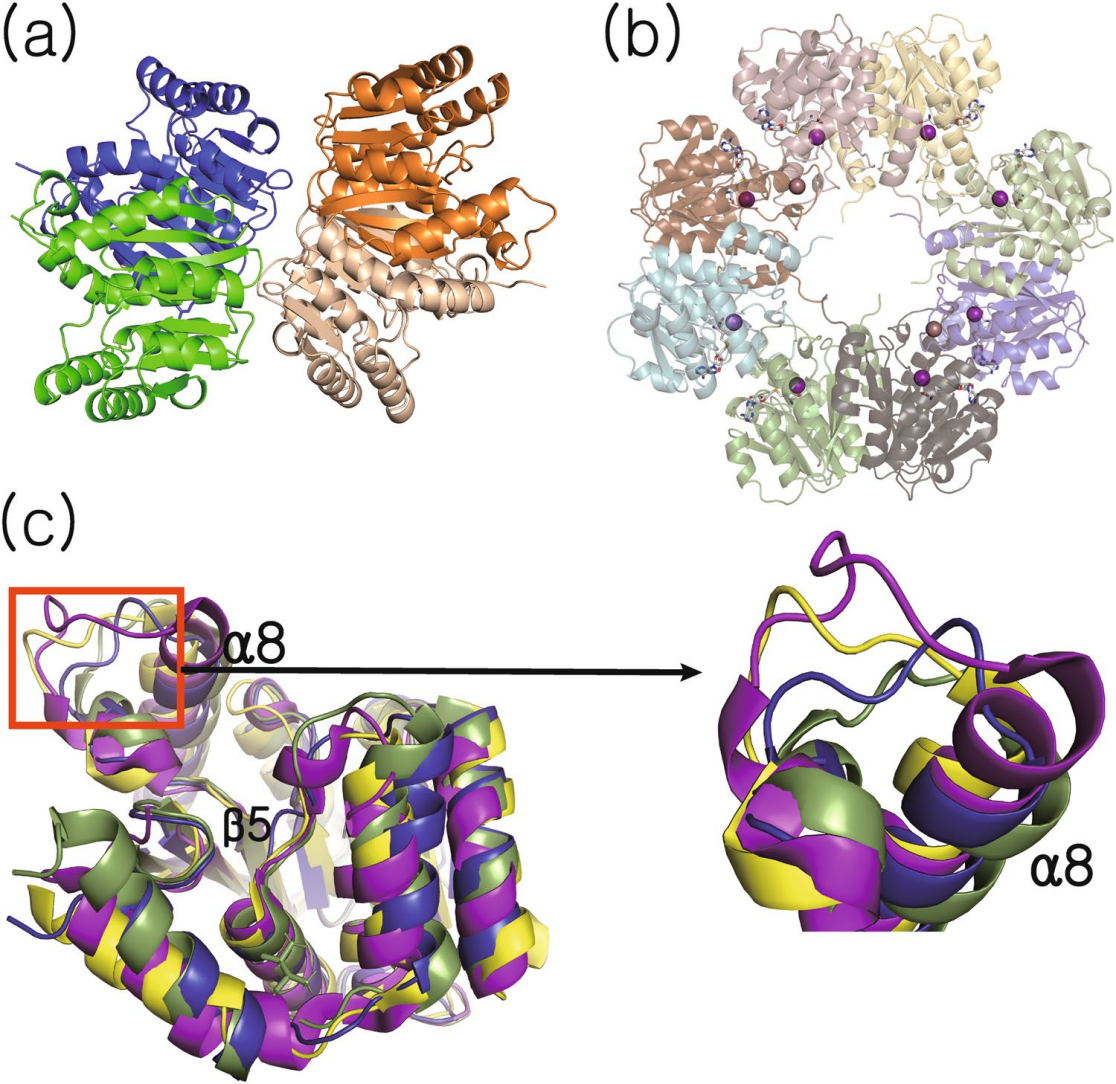
Overall structures of Rv0187. Ribbon representation of crystal structures of (**a**) ligand-free and (**b**) cofactor-bound forms of Rv0187, where SAH and the strontium ion are shown as white gray sticks and a purple sphere, respectively. Shown is the composition of the asymmetric unit, with four and eight copies of monomer in (**a**) and (**b**), respectively. (**c**) Conformational comparison of Rv0187 with its three closest structural homologs. Each monomer is superimposed, and ligand-free Rv0187 is presented in blue, the putative OMT of *C. glutamicum* (PDB code 3DR5) is yellow, *S. achromogenes* TomG (5N5D) is green, and *S. bicolor* CCoAOMT (5KVA) is purple. Inset is a magnified view of the region where the largest conformational diversity occurs.

X-ray crystal structures of the apo-form (hereafter denoted as ‘ligand-free structure’) and of a complex with Sr^2+^ and S-adenosyl-l-homocysteine (SAH) (hereafter denoted as ‘cofactor-bound structure’) have been determined to a resolution of 2.08 and 1.64 Å, respectively. Crystallographic statistics are summarized in Table 1. An asymmetric unit (ASU) of the ligand-free structure contains four copies of the monomer (Fig. 2a). Meanwhile, the ASU of the cofactor-bound structure is composed of eight monomers, and each subunit exhibits the unambiguous presence of a divalent metal ion and SAH (Fig. 2b). The overall conformation of ligand-free and cofactor-bound forms is highly similar, with an average Cα rmsd of 0.347 Å between monomers. The protein adopts the Rossmann fold typical for numerous SAM-dependent methyltransferases, containing seven core β-sheets surrounded by eight α-helices (α_1_α_2_α_3_β_1_α_4_β_2_α_5_β_3_α_6_β_4_α_7_β_5_α_8_β_6_β_7_). A search for structural homologs of the ligand-free structure of Rv0187 identified *Streptomyces achromogenes* TomG as the top hit (PDB code 5N5D, Z-score 31.8, rmsd 1.7 Å), followed by caffeoyl-CoA OMT from *Sorghum bicolor* (PDB code 5KVA, Z-score 25.6, rmsd 2.1 Å), and a putative OMT from *Corynebacterium glutamicum* (PDB code 3DR5, Z-score 25.2, rmsd 2.1 Å)^22^. When the structures of these top three hits are superimposed with that of the ligand-free structure of Rv0187, the largest conformational diversity is observed around a loop connecting β5 and α8 of MTB Rv0187, defined by a stretch of amino acid residues, Asn-166 through Ala-179 (Fig. 2c). In both ligand-free and cofactor-bound structures of Rv0187, the loop extends straight from the metal and SAM/SAH binding site towards the solvent channel.

**Table 1:** Crystallographic statistics.

	ligand- free	Cofactor-bound
**Data collection**		
Space group	P 2_1_ 2_1_ 2_1_	P 2_1_ 2_1_ 2_1_
**Cell dimensions**		
*a*, *b*, *c* (Å)	81.33 94.26 125.68	75.30 75.92 329.84
Resolution (Å)	50.0–2.08 Å (2.12–2.08 Å)	62.47–1.64 Å (1.67–1.64 Å)
*R*_merge_	0.243 (1.75)	0.148 (2.44)
	17.4 (2.45)	13.4 (1.2)
Completeness (%)	98.6 (96.9)	98.9 (92.7)
Redundancy	7.0 (6.1)	14.4 (12.4)
**Refinement**		
Resolution (Å)	29.24–2.08 Å	36.23–1.64
No. reflections	57,797	227,000
*R*_work_/*R*_free_	0.203/0.231	0.181/0.207
**No. atoms**		
Protein	6276	12824
Ligand	16	291
Water	106	873
**B-factors (Å** ^**2**^ **)**		
Protein	29.5	35.3
ligand	29.6	31.9
Water	25.8	39.5
**R.m.s deviations**		
Bond lengths (Å)	0.010	0.007
Bond angles (°)	1.54	0.95
MolProbity validation		
Ramachandran favored	96.7	96.8
Ramachandran allowed	99.9	99.8
Ramachandran Outliers (%)	0.12	0.17
Molprobity score	1.80 (90^th^ percentile)	1.38 (94^th^ percentile)
Clashscore (all-atom clash score)	3.25	3.92

### Dimerization interface

In both ligand-free and cofactor-bound structures, Rv0187 appears to form a canonical dimer observed for all known COMT structures, consistent with the results of our SEC experiments. The buried surface areas at the dimerization interface are estimated to be 3,780 Å^2^ between A and B chains and 3,820 Å^2^ between C and D chains in the ligand-free structure^23^. Similar values are obtained for four biological dimers in the cofactor-bound structure, ranging from 3,796 to 3,854 Å^2^. Approximately 50 amino acid residues from each monomer participate in forming the homodimeric interface, which is distributed over α1, α5, α8, β6, β7, and a part of the β5-α8 loop. The electrostatic potential mapped on the surface of the ligand-free structure shows that the dimerization appears to be driven mostly by hydrophobic interactions, with eight residues from each monomer contributing to the formation of hydrogen bonds or salt-bridges within 3.2 Å, *i.e*., Pro-9, Asp-13, Gln-47, Lys-50, Asp-199, Thr-201, Ala-202, and Gln-204 (Supplementary Fig. 3).

### Cofactor binding sites

**Figure 3:**
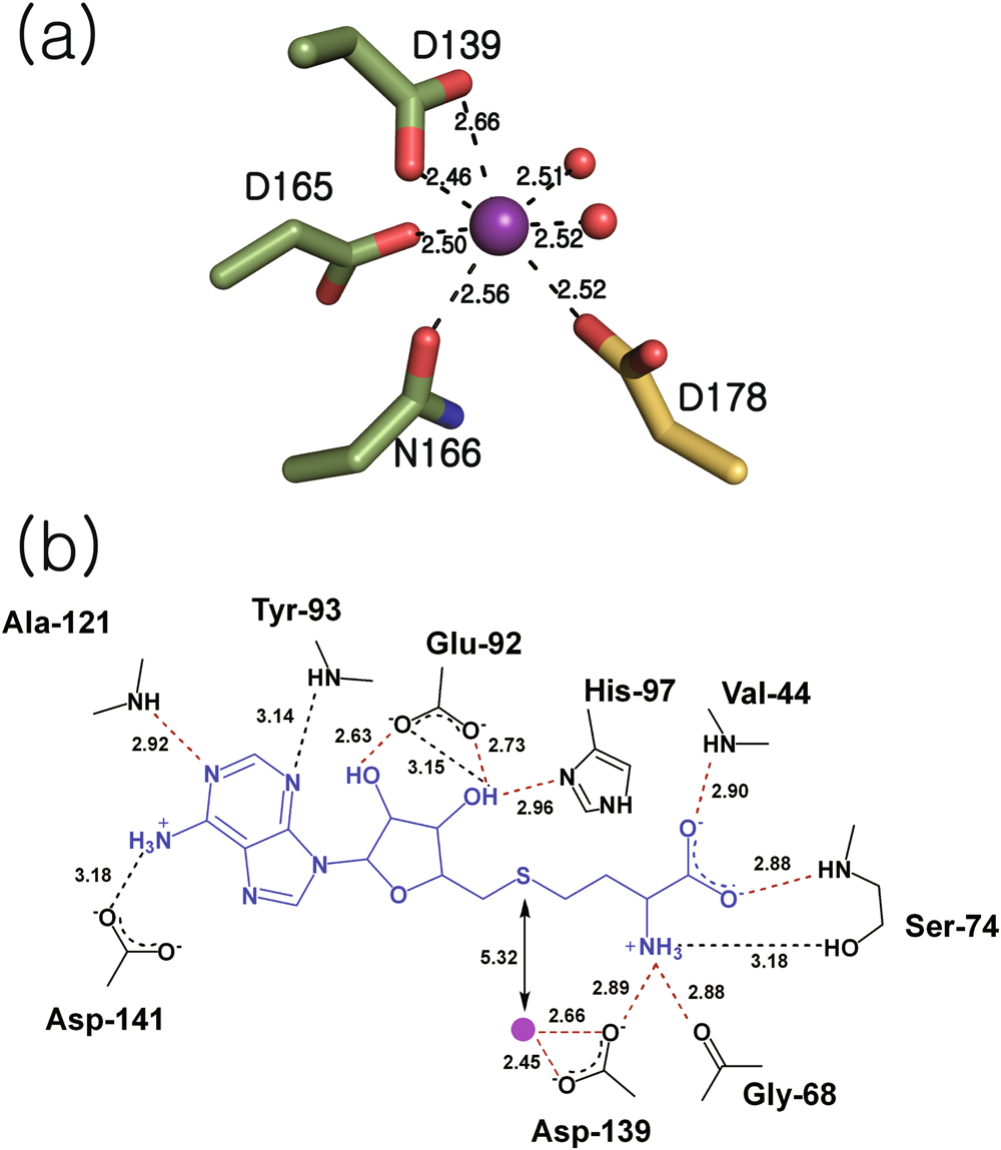
Molecular interactions between Rv1087 and cofactors. (**a**) A strontium ion (purple sphere) is coordinated by Asp-139, Asp-165, Asn-166 (green), two water molecules (red), and Asp-178 of a nearby subunit (yellow), which constitute the pseudobipyramidal metal coordination geometry. Metal-ligand distances averaged over eight subunits are labeled in Å. (**b**) Average distances of hydrogen bonds between SAH (blue) and amino acid residues (black) less than 3.2 Å in length are shown, where the distances of universally observed interactions in all eight chains are illustrated by red broken lines, and those observed in selective chains are black. The distance between the sulfur atom of SAH and the strontium ion (purple circle) is shown with solid black arrows.

At the initial stage of model building from X-ray diffraction data, excessive electron density around the modeled magnesium ion was observed in the *F*o-*F*c difference Fourier map (Supplementary Fig. 4), signifying the presence of a more electron-rich element despite magnesium salt being added to the protein sample. Since strontium chloride was included in the crystallization cocktail solution, the extra electron density most likely arose from Sr^2+^. Therefore, Sr^2+^ was included in the model for the subsequent refinement stages in place of Mg^2+^. Pseudobipyramidal geometry of strontium coordination is observed (Fig. 3a), similar to the octahedral coordination of magnesium or calcium in other COMT structures^4,13,15–17,19,24^. In the cofactor-bound structure of Rv0187, a strontium ion is complexed by Asp-139, Asp-165, Asn-166, two water molecules, and Asp-178 of an adjacent subunit, which is non-crystallographically related. The strontium ion is overall hepta-coordinated in all eight subunits of cofactor-bound structure, with Asp-139 interacting with the metal ion in a bidentate fashion.

Located close to the metal binding site is SAH, a product from COMT-dependent methyltransfer reactions, where the sulfur atom is approximately 5.3 Å distant from the Sr^2+^ (Fig. 3b). The overall binding pose of SAH is essentially identical, with slight variations in each subunit. The hydrogen-bonding interaction between the N1 of the adenine moiety and the backbone amide group of Ala-121 is universally conserved in all eight chains. Additionally, adenine forms a hydrogen bond with the backbone amide group of Tyr-93 through the N3 in chains E and F or with the side chain of Asp-141 through the N6 in chains D and G. The ribose 2′- and 3′-OH groups both interact with the side chain of Glu-92, and the 3′-OH is further hydrogen bonded to the imidazole side chain of His-97 in all monomers. The amine group of the methionyl moiety forms hydrogen bonds with the backbone carbonyl oxygen of Gly-68 and with the side chains of Asp-139 and Ser-74. The carboxyl group of SAH interacts with the protein via hydrogen bonding with the backbone amide of Val-44 in all monomers and with Ser-74 in chains A, B, and C (Fig. 3b and Supplementary Fig. 5).

### Active site conformation in the ligand-free structure

**Figure 4:**
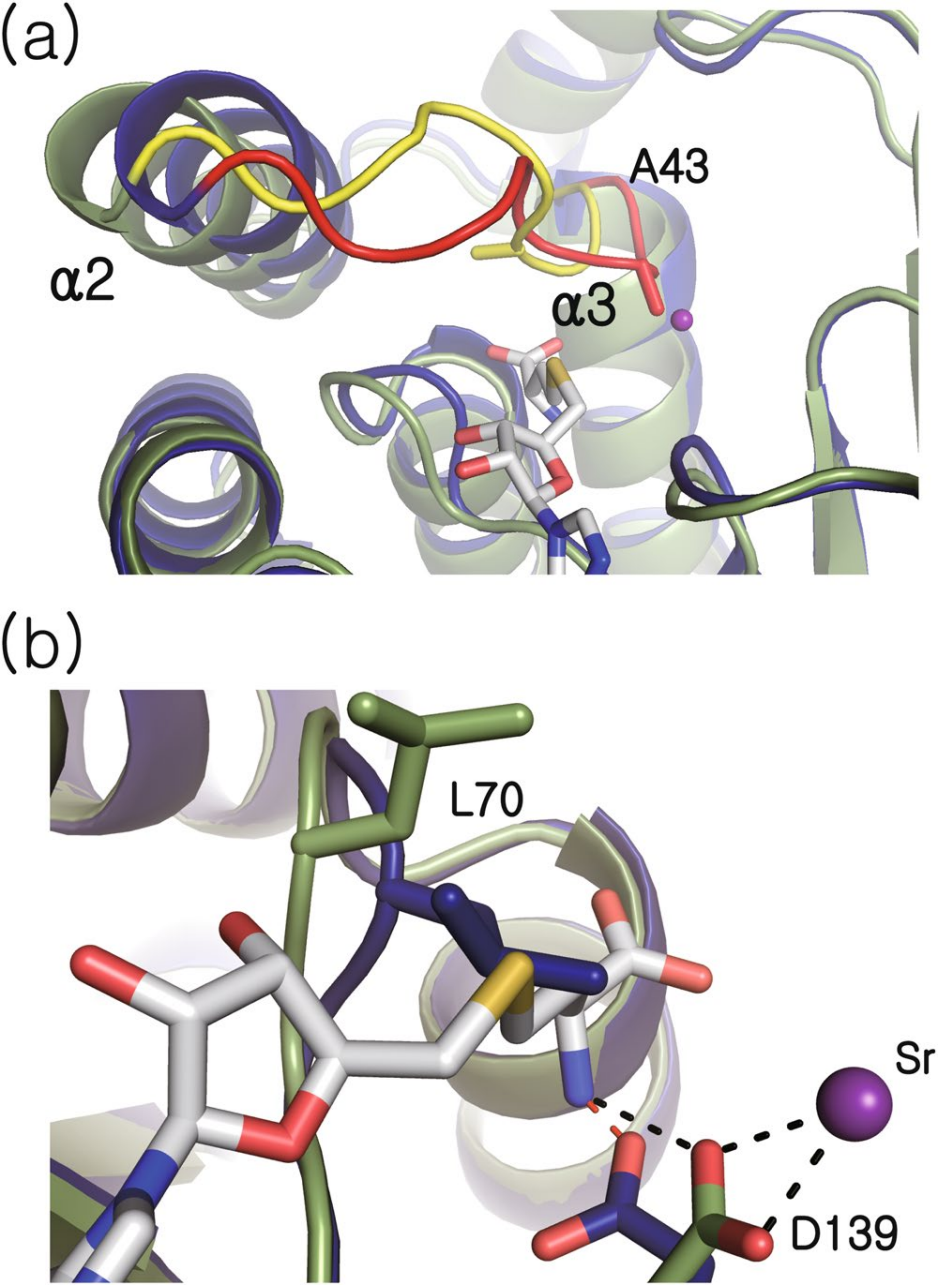
Comparison of ligand-free and cofactor-bound structures. (**a**) Ribbon representations of ligand-free and cofactor-bound forms are shown in blue and green, respectively. In the cofactor-bound structure, SAH and strontium ions are presented at white-gray sticks and a purple sphere, respectively. The loops connecting α2 and α3, which undergo the largest conformational change between ligand-free and cofactor-bound structures are highlighted in red and yellow, respectively. (**b**) Close-up view of superimposed ligand binding sites of ligand-free and cofactor-bound structures.

Compared to the cofactor-bound structure, the most notable conformational difference in the ligand-free structure is observed around the loop between α2 and α3 (amino acid residues Glu-38 through Ser-45), which restricts the ligand binding pocket (Fig. 4a). In particular, the Cβ of Ala-43 is shifted towards the metal binding pocket by approximately 6.2 Å, resulting in partial blockage. Furthermore, the rearrangement of the α2-α3 loop forces Leu-70 to flip, which causes the protrusion of the side chain into the binding pocket for the methionyl moiety of SAH (Fig. 4b and Supplementary Fig. 6). Another noteworthy conformational feature in the ligand-free structure is that the side chain of Asp-139 swings away from the divalent metal binding site and interacts with the backbone carbonyl groups of Ile-67 and Gly-68 instead. Overlaid with the SAH-bound structure, this conformation of the sidechain of Asp-139 in the ligand-free structure would be too close to the amine group of SAH (approximately 1.2 Å between the δO of Asp-139 and the N of SAH) and predicted to cause a clash upon binding of SAM/SAH.

### Isothermal titration calorimetric measurement of SAM binding

**Figure 5:**
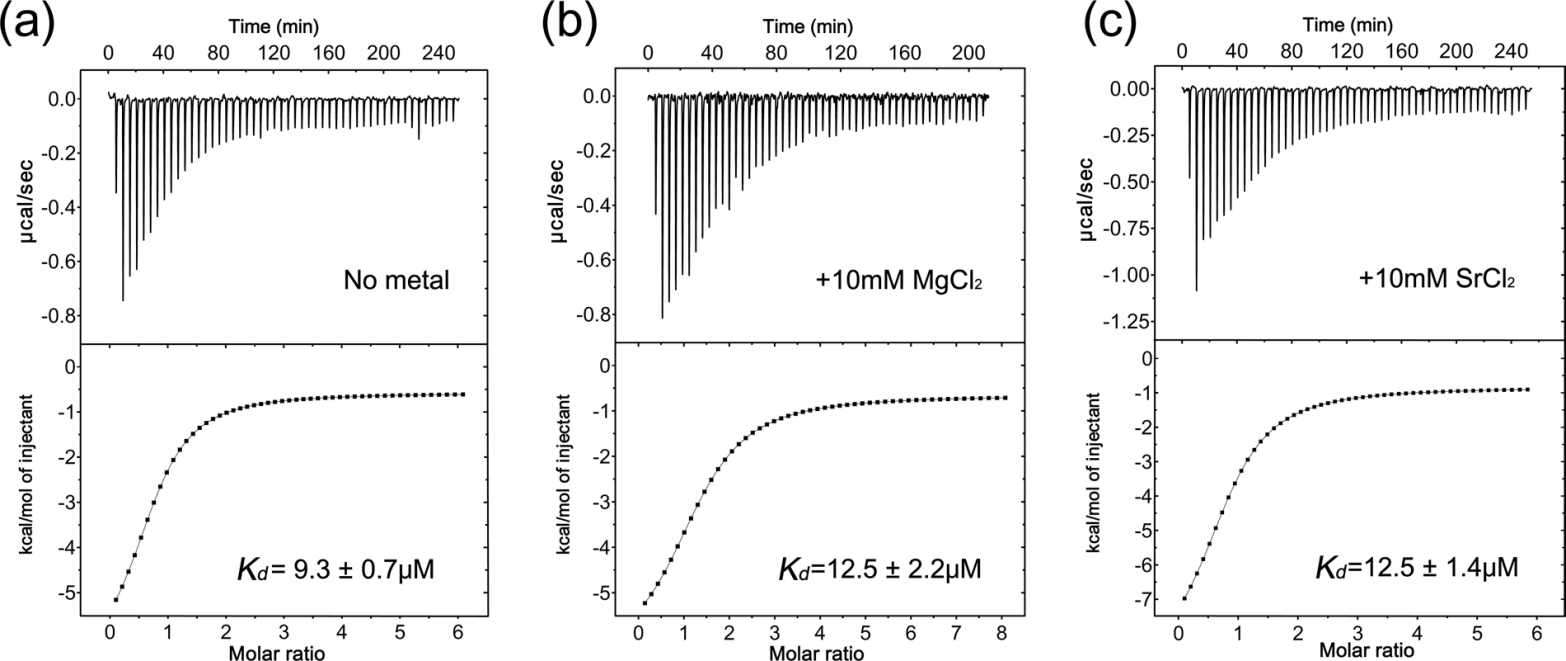
Isothermal titration calorimetry of Rv0187 with SAM. The SAM binding affinity as measured by ITC experiments in the absence (**a**) and in the presence of 10 mM magnesium chloride (**b**) or 10 mM strontium chloride (**c**). Representative plots of raw heat changes (top) and the normalized data (bottom) are shown. Dissociation constants (*K*_*d*_*)* and errors are averages and the standard errors from three independent experiments, respectively.

The thermodynamics of Rv0187 binding SAM were determined by isothermal titration calorimetry (ITC) in the absence or presence of 10 mM MgCl_2_ or SrCl_2_ (Fig. 5). The isotherms of the experiments were monophasic in both cases, indicating a single binding site of the enzyme for SAM, as predicted from crystal structures. The thermodynamic parameters for the interactions with SAM indicate that the binding process is primarily enthalpically driven in both cases (Supplementary Table 1). The thermodynamic interaction between Rv0187 and SAM is characterized by a dissociation constant (*K*_*d*_) of 9.3 ± 0.7 μM in the absence of a divalent metal ion, and similar parameters have been derived from data obtained in the presence of Mg^2+^ or Sr^2+^, with *K*_*d*_ = 12.5 ± 2.2 or 12.5 ± 1.4 μM, respectively.

### Analysis of copurified metabolite with the recombinant Rv0187

Naturally bound metabolites often offer direct insight into the biological function of the protein, which have originated from the expression host^25,26^. In an effort to identify a physiological substrate of MTB Rv0187, copurified ligands were extracted from the recombinant protein expressed in *E. coli* and analyzed with LC-MS (Supplementary Fig. 7). Compounds with mass-to-charge ratios (m/z) of 298.09 and 314.09 were detected, consistent with methylthioadenosine (MTA) (theoretical m/z = 298.097, calculated for C_11_H_16_N_5_O_3_S) and its oxidized form MTA-SO (theoretical m/z = 314.092, calculated for C_11_H_16_N_5_O_4_S), respectively. Further fragmentation analysis of the ligands by LC-MS/MS confirmed that the ligand contained adenine and ribose moieties, supporting our assignment. Notably, MTA and MTA-SO were detected in the protein sample prepared from the dimer peak of the size-exclusion chromatographic step but not in that from the higher-order oligomer peak. No additional ligand was detected at a significant level. Since MTA (and MTA-SO) is metabolically derived from SAM, we were unable to identify a candidate metabolite that may serve as a nucleophile for the Rv0187-dependent methyltransfer reaction in MTB.

### *In vitro* activity and regioselectivity on catechol-like metabolites

**Figure 6:**
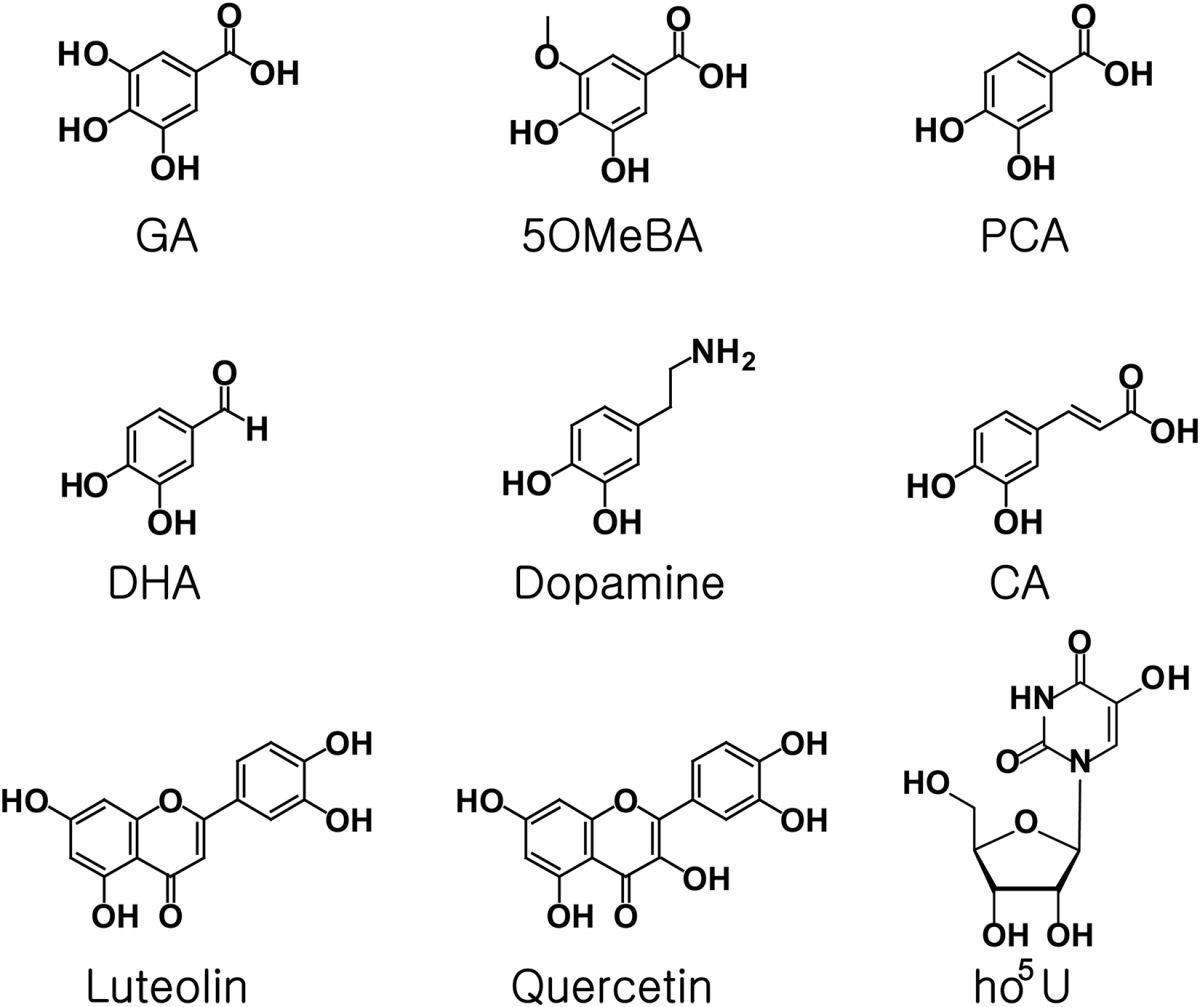
Chemical structures of catechol-like compounds tested as substrates for methyltransfer activity of Rv0187. Catechol-containing metabolites were screened with recombinant Rv0187 for methyltransfer reactions using HPLC or LC-MS. GA, gallic acid; 5OMeBA, 3,4-dihydroxy-5-methoxy-benzoic acid; PCA, protocatechuic acid; DHA, 3,4-dihydroxy-benzaldehyde; CA, caffeic acid; ho^5^U, 5-hydroxyuridine.

**Figure 7:**
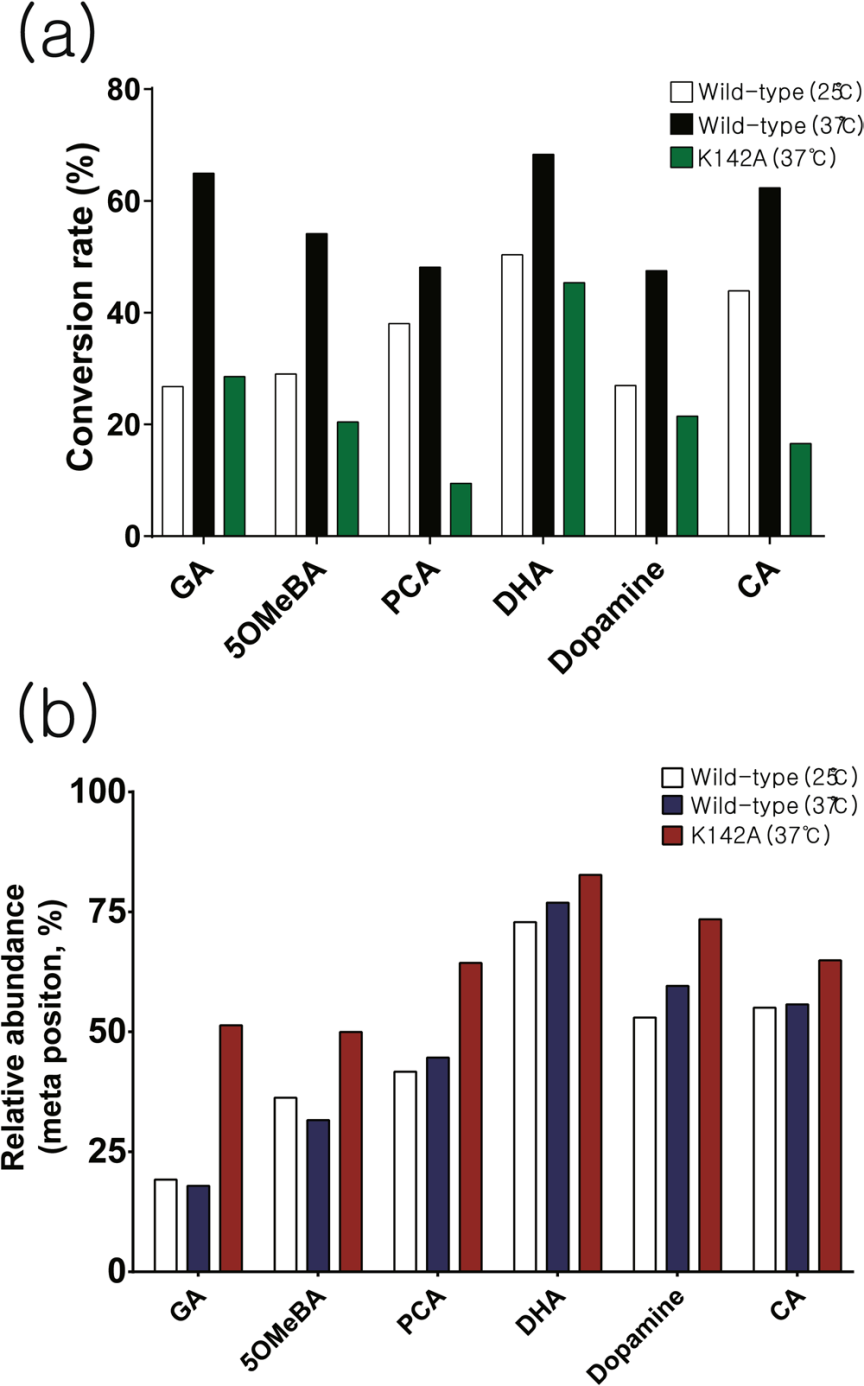
*In vitro* activity and regioselectivity of Rv0187. (**a**) Apparent conversion rates of selected substrates by the wild-type Rv0187 at 25 °C (white bars) and 37 °C (black bars). Green bars represent conversion rates using the K142A mutant enzyme at 37 °C. (**b**) Relative formation of meta isomers is shown, where white, navy and red bars represent the relative amount of a meta-methylated compound produced by the wild type at 25 and 37 °C and by K142A at 37 °C, respectively.

Previously reported metabolites subject to methyltransfer by various COMTs were analyzed for *in vitro* activity with Rv0187 to determine the scope of substrate specificity. A total of nine compounds with a catechol moiety were tested, and the SAM-dependent methyltransfer activity was verified by HPLC (Fig. 6 and Supplementary Fig. 8) and LC-MS analyses (Supplementary Fig. 9), suggesting a broad substrate specificity of Rv0187. A mixture of both regioisomers of mono-methylated products (meta- and para-methoxy compounds) has been detected from reaction mixtures of gallic acid (GA), 3,4-dihydroxy-5-methoxy-benzoic acid (5OMeBA), protocatechuic acid (PCA), 3,4-dihydroxy-benzaldehyde (DHA), dopamine, and caffeic acid (CA). Meanwhile, methylation of 5-hydroxyuridine (ho^5^U) yielded a single product, 5-methoxyuridine (mo^5^U). In addition, Rv0187 was able to methylate flavonoids (luteolin and quercetin), although the precise methylation site could not be assigned. Next, we measured the overall conversion rate, i.e., the proportions of total isomeric products from a substrate, at 25 and 37 °C. The enzyme exhibited a slightly higher activity at 37 °C than at 25 °C overall; 25–50% of substrates were turned over at 25 °C, whereas 50–65% of substrates were turned over at 37 °C. Since Lys-142 has been predicted to be a general base from previous studies of other COMTs^16,27,28^, we have examined the catalytic activity of a mutant enzyme where the lysine has been replaced with alanine (K142A). Unexpectedly, the mutation did not severely hamper O-methyltransfer activity, and the mutant enzyme retained a lower yet substantial activity compared to that of the wild type. The formation of each regioisomer was quantitatively measured under two different temperature conditions (Fig. 7 and Supplementary Table 2). DHA, dopamine and CA were preferentially methylated at the meta position, whereas PCA, 5OMeBA, and GA were more readily modified at the para-hydroxyl group. Although temperature variation did not affect regioselectivity of the enzyme, alanine mutation of Lys-142 *did* alter the methylation pattern by enhancing overall methylation of the meta hydroxyl group, which led to minimal regioselectivity against GA and 5OMeBA, reversal of selectivity with PCA, or further increased meta preference with DHA, dopamine and CA.

### Analysis of steady-state kinetics

First, the metal dependency of the enzyme was tested and confirmed, where the addition of EDTA to the assay mixture completely abolished the activity (Supplementary Fig. 10A). We exploited this property as a way of terminating the enzymatic activity and performed initial velocity experiments at various concentrations of a catechol substrate at saturating levels of SAM for 5OMeBA, PCA and CA (Supplementary Fig. 10B and Supplementary Table 3). The enzymatic reaction was most efficient with 5OMeBA (*K*_*M*_ and *k*_*cat*_ of 0.030 ± 0.004 mM and 0.080 ± 0.004 min^−1^, respectively). For PCA, kinetic parameters of *K*_*M*_ = 0.15 ± 0.02 mM and *k*_*cat*_ = 0.22 ± 0.01 min^−1^ were obtained, whereas those for CA were *K*_*M*_ = 0.49 ± 0.17 mM and *k*_*cat*_ = 0.43 ± 0.07 min^−1^.

## Discussion

We have determined two crystal structures of MTB Rv0187 (UniProt ID: O07431), one with and one without cofactors; this enzyme has been annotated as a probable O-MTs. MTB encodes two additional O-MTs, Rv1220 and Rv1703, and Rv1220 does not bind a divalent metal ion, unlike other Class I COMTs. Sequence neighbors of MTB Rv0187 within the O-methyltransferase family (methyltransf_3; PF01596) were identified by constructing a sequence similarity network (SSN) in an effort to predict the biological function (Supplementary Fig. 11). Separate from the clusters of mammalian COMTs or plant CCoAOMTs, MTB Rv0187 belongs to a group mostly composed of bacterial proteins, including *S. achromogenes* TomG, *Klebsiella pneumonia* OMT, and *Bacillus cereus* OMT (BcOMT2), structures of which have been reported. The physiological functions of the last two are currently unknown, although it was demonstrated that *B. cereus* OMT was able to methylate selected flavonoid compounds *in vitro*^27^. TomG is a member of the proteins required for the assembly of the antibiotic/antitumor agent tomaymycin^9,21^, sharing 54% sequence identity with MTB Rv0187. Moreover, the structure of TomG is the most homologous to that of Rv0187 among all known structures as described in the Results, implying a significant similarity of the biological targets of these two enzymes. TomG has been proposed to methylate the 5-hydroxyl group of 4,5-dihydroxyanthranilic acid or its thioester form with TomA *in vivo*, and *in vitro* methylation of the free acid was verified. Therefore, it is likely that Rv0187 acts on compounds with a chemical structure similar to that of 4,5-dihydroxyanthranilic acid, although it is not known whether MTB produces tomaymycin or a related compound. Unfortunately, we were not able to test the activity of Rv0187 on 4,5-dihydroxyanthranilic acid because the acid’s poor stability hampered our attempted chemical synthesis. However, the steady-state kinetics demonstrate that a structurally similar compound, such as 5OMeBA, is reasonably well accepted by Rv0187 with a *k*_*cat*_*/K*_*M*_ of 2.7 × 10^3^ min^−1^·M^−1^, which is comparable to the values found for other COMTs^10,17,29^.

We analyzed copurified metabolites with recombinant Rv0187 expressed in *E. coli* to gain insights into the physiological substrate. Two ligands were detected in the sample, MTA and MTA-SO. The latter is an oxidized form of MTA and has been identified in human urine, especially at elevated levels from breast cancer patients or those suffering severe immunodeficiency^30,31^. The origin of this metabolite has not yet been established, although it was shown that air oxidation of MTA could lead to MTA-SO^30^. To our knowledge, this metabolite has not been identified in bacteria, and we speculate that the compound is likely formed from nonenzymatic oxidation of MTA during sample preparation. Because MTA or MTA-SO has been derived from SAM, no meaningful clue to the identity of a potential electrophile could be obtained from the present data. It is possible that the binding affinity of a putative ligand to the recombinant protein is not strong enough to survive the rigorous washing steps before extraction or that the true biological substrate is simply not present in the metabolic pool of *E. coli*, which remains to be discovered.

Recombinant Rv0187 has been shown to methylate a variety of natural products with vicinal hydroxyl groups *in vitro* (Supplementary Fig. 8). Broad substrate specificity allows the enzyme to act on flavonoids (quercetin and luteolin) and 5-hydroxyuridine (ho^5^U), a modified nucleoside. Our laboratory has recently shown that *B. subtilis* TrmR directs methylation on the 5-hydroxyl group of ho^5^U at the wobble position of tRNA in the biosynthesis of 5-methoxyuridine, a modified nucleoside widespread among gram-positive bacteria^12^. We examined the cross-activity of these enzymes but could not detect a significant activity of TrmR on ho^5^U or of Rv0187 on ho^5^U-containing tRNA (data not shown). Another noteworthy functional feature of Rv0187 is that regioselectivity appears to be less stringent for it than for eukaryotic COMTs; the enzyme exhibits a meta preference towards CA, dopamine, and DHA, while it prefers to methylate the *para*-OH of GA, 5OMeBA, and PCA. COMTs contain a unique ‘insertion loop’ (β5-α8 loop in Rv0187), which is a part of the substrate binding pocket and serves as a determinant for a catechol substrate. The amino acid composition of an insertion loop is highly variant among COMTs (Supplementary Fig. 1). Structural comparison of insertion loops of RnCOMT, *M. sativa* CCoAOMT, and *S. achromogenes* TomG with that of Rv0187 immediately reveals a significant difference in shapes and lengths. Furthermore, the insertion loop is often disordered in several crystal structures of bacterial COMTs, including MTB Rv1220 (pdb code 5  X7 F), *B. cereus* OMT (3DUL), *B. subtilis* TrmR (5ZW3), *Geobacter sulfurreducens* OMT (3C3P), and *Bacillus halodurans* OMT (2GPY). Interestingly, binding of a substrate allows a structural transition of the insertion loop to an ordered state in cases of *B. cereus* OMT (3DUW)^27^ and *B. subtilis* TrmR (5ZW4)^12^, underscoring the dynamic nature of substrate binding by COMTs. The sequence diversity and structural flexibility of the insertion loop suggest a wide range of biological activities in the COMT family.

In the ligand-free structure Rv0187, the side chain of Leu-70 extends into the SAM binding pocket. However, this residue is flipped over to accommodate the binding of SAH in the cofactor-bound structure. Similar trends have been found in other COMT structures, *e.g*., an equivalent role is played by Tyr-75 of PaMTH1^4^ and Leu-68 of *B. cereus* OMT^27^. Therefore, an induced-fit binding employing a bulky hydrophobic residue near the SAM binding cavity may be a more general theme conserved among the COMT family than previously recognized. These enzymes absolutely require divalent metal ions for activity, as Rv0187 does. A possibility exists where sequestration of metal induces conformational change around the active site, controlling the binding of SAM for these enzymes. The presence of MgCl_2_ indeed enhanced the affinity for SAM, where dissociation constants (*K*_*d*_) decreased from 113 to 21 μM for PaMTH1^4^. However, this hypothesis is likely to be ruled out for Rv0187, since dissociation constants for SAM in the presence (*K*_*d*_ = 12.5 μM) or absence of metal ions (*K*_*d*_ = 9.3 μM) do not change significantly indicating that affinity for SAM is independent of the active-site metal ion. Other known class I COMTs exhibit similar *K*_*d*_ values for SAM, *e.g*., 8.6 μM for SbCCoAMT from *S. bicolor* and 13.6 μM for spCOMT from *Schizosaccharomyces pombe*^15,32^.

Lys-142 of Rv0187 is a highly conserved residue among the COMT family (96.4% conservation) and has been proposed to be a general base^28^. However, the alanine mutant enzyme was still able to methylate catechol compounds at a rate comparable to that of the wild type while displaying slightly enhanced methylation of *meta*-OH. These results support a model in which Lys-142 of Rv0187 does not activate the nucleophilic hydroxyl group but is engaged in substrate binding. Similarly, the mutation of Lys-144 of RnCOMT, which is equivalent Lys-142 of Rv0187, did not abolish RnCOMT activity^33^. The authors proposed that a solvent molecule is capable of deprotonating the nucleophile, which is depolarized by the bound metal. Currently, it is not clear which residue is responsible for the deprotonation of a catechol substrate in Rv0187, and further biochemical/structural studies are required to understand the enzymatic mechanism of the methyltransfer reaction.

## Methods

### Cloning and purification of MTB Rv0187

The Rv0187 gene was amplified from the genomic DNA of *Mycobacterium tuberculosis (H37Rv*) by polymerase chain reaction (PCR) using the following primers and inserted into the pLATE51 or pLATE31 expression vector using the LIC Cloning kit (Thermo Scientific): For the N-terminal His6-tagged construct (pLATE51-Rv0187), pLATE51F (GGTGATGATGATGACAAGATGGACCAGCAACCCAACCCGCCC) and pLATE51R (GGAGATGGGAAGTCATTACCGCACCAAAGCGAGGGCGAAACC) were used. For the C-terminal His6-tagged construct (pLATE31-Rv0187), pLATE31F (AGAAGGAGATATAACTATGGACCAGCAACCCAA) and pLATE31R (GTGGTGGTGATGGTGATGGCCCCGCACCAA) or pLATE31-TEV-R (GTGGTGGTGATGGTGATGGCCGCCTTGGAAGTATAAGTTCTCCCGCACCAAAGC) for an additional TEV cleavage site (pLATE31-Rv0187-TEV) were used.

Single alanine mutation of Lys-142 (K142A-Rv0187) was introduced by the QuikChange II XL Site-Directed Mutagenesis Kit (Agilent) using a pair of primers: GTGTTCATCGACGCCGACGCAGAGAACAACGTCGCATA and TATGCGACGTTGTTCTCTGCGTCGGCGTCGATGAACAC. Expression plasmids containing the Rv0187 gene were selected by the ampicillin marker, and the sequence of the inserted gene was verified by DNA sequence analysis (Macrogen, Korea).

*Escherichia coli* BL21(DE3) cells were transformed with a recombinant DNA plasmid harboring the Rv0187 gene and grown in LB containing 100 mg/ml ampicillin at 37 °C. Overexpression of the gene product was induced with 0.5 mM isopropyl-D-1-thiogalactopyranoside (IPTG) at OD_600_ = 0.5. Cells were incubated overnight (>16 h) at 20~25 °C and harvested by centrifugation. The cell pellet was resuspended in lysis buffer (25 mM HEPES, 150 mM KCl, pH 7.5, and 2 mM dithiothreitol (DTT)) followed by lysis using sonication for 15 min. The lysate was centrifuged at 18000 g, and the supernatant was applied to a Ni-NTA (HisTrap HP, GE Healthcare) column and eluted with a linear gradient of imidazole (10~500 mM). The buffer for the elution step was the same as the lysis buffer minus DTT. Gel filtration using a Superdex 75 pg column (GE Healthcare, USA) was used for further purification. To calculate the concentration of a purified protein, the following extinction coefficients for Rv0187 were used: ε280 = 20.9 cm^−1^ mM^−1^ for recombinant Rv0187 with a TEV cleavage site and 19.5 cm^−1^ mM^−1^ for the other constructs.

### Chemicals

Gallic acid (GA), *trans-*caffeic acid (CA) and its methylated products (3-hydroxy-4-methoxycinnamic acid (trans), *trans*-ferulic acid), 3,4-dihydrobenzaldehyde (DHA) and its methylated products (vanillin, iso-vanillin), protocatechuic acid (PCA), dopamine hydrochloride and its methylated products (3-hydroxy-4-methoxy-phenethylamine hydrochloride, 3-methoxy-tyramine hydrochloride), quercetin, and luteolin were obtained from Sigma-Aldrich (USA). Two major methylated products of GA (3,4-dihydroxy-5-methoxybenzoic acid (5OMeBA), 3,5-dihydroxy-4-methoxybenzoic acid), methylated products of 5OMeBA (5-hydroxyveratric acid, syringic acid), methylated products of PCA (3-hydroxy-4-methoxybenzoic acid, 4-hydroxy-3-methoxybenzoic acid), 5-hydroxyuridine (ho^5^U) and the methylated product 5-methoxyuridine (mo^5^U) were purchased from Carbosynth (UK).

### LC-MS-based *in vitro* methyltransfer assay

The Rv0187-dependent methylation activity was assayed by adding 10 μM enzyme (purified using the pLATE31-Rv0187 construct) to a mixture of 25 mM Tris-HCl (pH 7.5), 100 mM NaCl, 3 mM MgCl_2_, 1 mM SAM, and 1 mM catechol substrate. For quercetin and luteolin, 150 μM each was used in the activity assay. The reaction was stopped by adding 10 mM ethylenediaminetetraacetic acid (EDTA) after 16 h, and each assay was performed in triplicate. An aliquot of 20 μl of the reaction sample was injected into a 6545XT AdvancedBio Agilent LC-QTOF-MS system coupled to a reverse phase HPLC column (Agilent C18 Column 100 Å, 3.5 μm, 4.6 mm × 150 mm) with a 0.3 ml/min flow rate. For positive ion mode, buffer A (DW + 0.1% (v/v) formic acid (FA)) and buffer B (acetonitrile (ACN) + 0.1% FA) were used as mobile phases, and for negative ion mode, buffer C (10 mM ammonium acetate) and buffer D (100% ACN) were used as mobile phases. Each compound was analyzed using different mobile phases and methods. For caffeic acid, a gradient of 0~3 min 95% (C) and 5% (D) to 100% (D) at 15 min was used; for dopamine, 0~3 min at 95% (C) and 5% (D) to 100% (D) at 30 min; for 3,4-dihydroxy-5-methoxy-benzoic acid, quercetin and luteolin, 0~3 min at 95% (A) and 5% (B) to 100% (B) at 15 min; for gallic acid, 0~3 min at 95% (A) and 5% (B) and 3~15 min at 75% (A) 25% (B) to 100% (B) at 20 min; for protocatechuic acid and 3,4-dihydroxy-benzaldehyde, an identical method as gallic acid was used. The initial velocities of the enzymatic reaction were measured by an HPLC instrument connected to a UV-spectrometer. Assays were initiated by adding 1 μM Rv0187 to a reaction mixture composed of 25 mM Tris-HCl (pH 7.5), 100 mM NaCl, 3 mM MgCl_2_, 1 mM SAM and varying amounts of a substrate, which was periodically quenched with 10 mM EDTA. The assay solution was injected into the reverse phase HPLC column, and the relative amount of a substrate and a product was estimated via peak integration. Steady-state kinetic parameters (*k*_*cat*_ and *K*_M_) were derived from fitting assay data to the Michaelis-Menten equation using GraphPad Prism7.

### Isothermal titration calorimetry

Titration experiments under three different conditions were performed at 25 °C using a Nano-ITC (TA Instruments). A buffer containing 20 mM HEPES (pH 7.5) and 50 mM KCl was used for titrations without divalent metal ions, whereas a buffer containing an additional 10 mM MgCl_2_ or 10 mM SrCl_2_ was used for titrations for divalent metal-dependent SAM binding titrations. The enzyme (38 or 48 μM) was titrated with 1 mM SAM in 50 steps (1 step = 5 μl). Raw data from ITC experiments were analyzed by NanoAnalyze software. Thermodynamic parameters were obtained from three independent experiments for each set of conditions.

### Quantitative estimation of regioisomeric excess

The assay mixtures (20 μl) were loaded onto a reverse phase HPLC column (Agilent C18 Column 100 Å, 3.5 μm, 4.6 mm × 150 mm) coupled to an Agilent 1200 HPLC system (Agilent) to resolve regioisomeric products, which were detected at λ = 280 nm (DHA, dopamine hydrochloride and their product), 260 nm (ho^5^U, PCA, GA, 5OMeBA, their products and two flavonoids (quercetin, luteolin)), or 320 nm (*trans-*caffeic acid and its products). The regioisomeric excess was evaluated using the relative peak area of each product.

### Extraction of naturally bound ligand from recombinant Rv0187

Naturally copurified ligand with the recombinant protein was extracted as described previously^25,26^ with a minor modification. In brief, a highly purified protein sample was concentrated to 10 mg/mL and then denatured by repeated cycles of incubation in liquid nitrogen for 1 min immediately followed by incubation in a heat block (95 °C) for 0.5~1 min until transparent protein solution became opaque from rapid aggregation. Aggregated protein samples were mixed with methanol (>95% (v/v)) and centrifuged at 17000 g for 30 min at 4 °C. The supernatant was collected and evaporated in CentriVap Benchtop Vacuum Concentrators (LABCONCO) coupled to a −84 °C Centrivap Cold Trap (LABCONCO) at 40 °C overnight. The sample was resuspended in water and analyzed with LC-MS and LC-MS/MS.

### Crystallization and structure determination of ligand-free Rv0187

First, 10 mg/mL Rv0187 fused with the N-terminal His6-tag (pLATE51-Rv0187 construct) was mixed with 2.8 M sodium acetate trihydrate, pH 7.4 (Hampton Research, USA), at a 1:1 ratio (v/v) and crystallized by the sitting drop method at room temperature. Protein crystals were obtained in 1~2 weeks. Crystals were harvested and cryoprotected with 20% glycerol, flash frozen and stored in liquid nitrogen. Diffraction data were collected at Pohang Accelerator Laboratory (PAL, 7 A Structural Biology beam line) and processed with the HKL2000 package^34^. The single wavelength λ = 0.9793 Å was used to produce diffracted images at 100 K, which were consistent with the space group P2_1_2_1_2_1_ with a unit cell of dimensions *a* = 81.3 Å, *b* = 94.3 Å, and *c* = 125.7 Å. Molecular replacement was performed using the structure of BcOMT2 from *Bacillus cereus*^27^ (PDB ID: 3dul) as the search model with MOLREP^35^. Subsequent model building and refinement were performed with Coot^36^ and REFMAC5^37^. The final model was refined to 2.08 Å with *R*_work_ and *R*_free_ values of 0.203 and 0.231, respectively. Ramachandran analysis indicates that 96.7% of residues are in favorable regions, 3.2% in allowed regions and 0.12% in outlier regions^38^.

### Crystallization and structure determination of cofactor-bound Rv0187

For cocrystallization, the recombinant Rv0187 fused with the C-terminal His6-tag after a TEV cleavage site (pLATE31-Rv0187-TEV construct) was used, where a protein–ligand mixture containing 0.22 mM Rv0187, 2 mM SAH and 2.6 mM MgCl_2_ was incubated at 4 °C for 1 h, mixed with a solution of 0.1 M HEPES (pH 7.3), 70% v/v (+/−)-2-methyl-2,4-pentanediol (MPD), and 0.1 M strontium chloride hexahydrate (Hampton Research, USA) at a 1:1 ratio (v/v) and crystallized as described for ligand-free Rv0187. The cocrystals were obtained in 3 days. Crystals were harvested, flash frozen and stored in liquid nitrogen. No additional cryoprotectant was used. Diffraction data were collected at Pohang Accelerator Laboratory (PAL, 7 A Structural Biology beam line) using a wavelength λ = 0.9793 Å at 100 K. Diffraction images were analyzed with iMosflm^39^ and were consistent with the space group P2_1_2_1_2_1_ with a unit cell of dimensions *a* = 75.3 Å, *b* = 75.9 Å, and *c* = 329.8 Å. Molecular replacement was performed with Molrep using the ligand-free structure of Rv0187 as a search model followed by model building and refinement with Coot and Phenix^36,40^. The final model was refined to 1.64 Å with *R*_work_ and *R*_free_ values of 0.181 and 0.207, respectively. Ramachandran analysis revealed that 96.8% of residues are in favored regions, 3.0% are in allowed regions, and 0.17% are in outlier regions^38^.

## Supplementary information

Supplementary Information
