# Supplementary material for: Structural and biochemical characterization of Rv0187, an O-methyltransferase from Mycobacterium tuberculosis
Source: Sci Rep. 2019 May 30;9:8059. doi: 10.1038/s41598-019-44592-7 (PMC6543040; doi:10.1038/s41598-019-44592-7)
Supplement: Supplementary file 1 — Supplementary Information [file 41598_2019_44592_MOESM1_ESM.docx]

**Supplementary Information**

**Structural and biochemical characterization of Rv0187, an O-methyltransferase from *Mycobacterium tuberculosis***

Sanghyun Lee**,** Jihoon Kang and Jungwook Kim^*^

Department of Chemistry, Gwangju Institute of Science and Technology, Gwangju, 61005, Korea. ^*^Corresponding author (jwkim@gist.ac.kr).


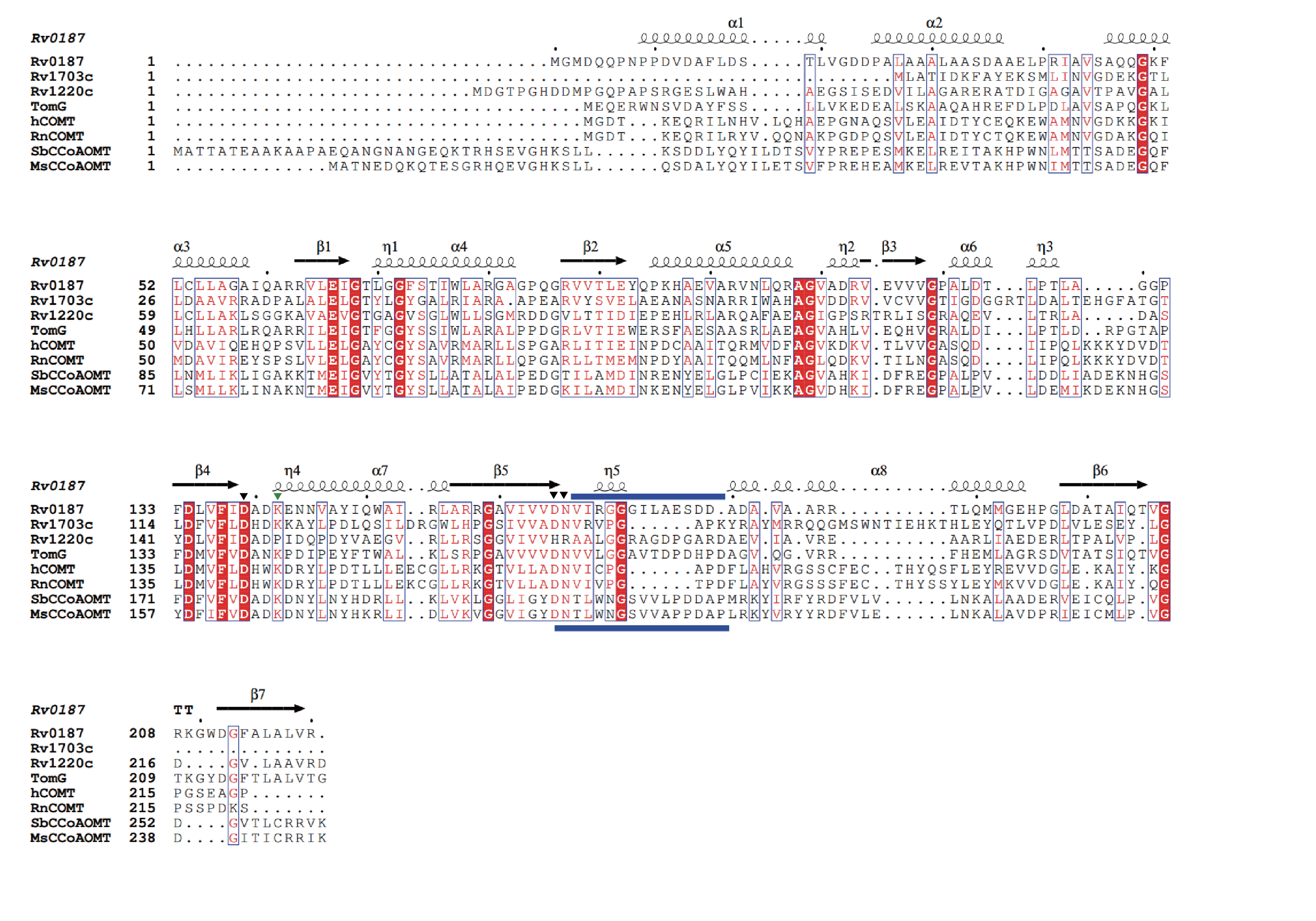


**Supplementary Figure 1. Multiple sequence alignment of Rv0187 with other OMTs.**

The amino acid sequence of Rv0187 (UniProtKB : O07431) is aligned with those of other representative Class I COMTs including Rv1703c (L0TAD5), Rv1220c (P9WJZ7) from *Mycobacterium tuberculosis*, TomG (A0A0J8AIC3) from *Streptomyces regensis*, hCOMT (P21964) from *Homo sapiens*, RnCOMT (P22734) from *Rattus norvegicus*, SbCCoAOMT (C5Z4W3) from *Sorghum bicolor* and MsCCoAOMT (Q40313) from *Medicago sativa*. Black triangles represent metal binding residues and the green triangle denotes a putative catalytic base in class I COMT. Insertion loop region is labeled with blue bars (amino acids 166 through 179 in Rv0187). The figure was prepared with ESPript 3.0^1^.


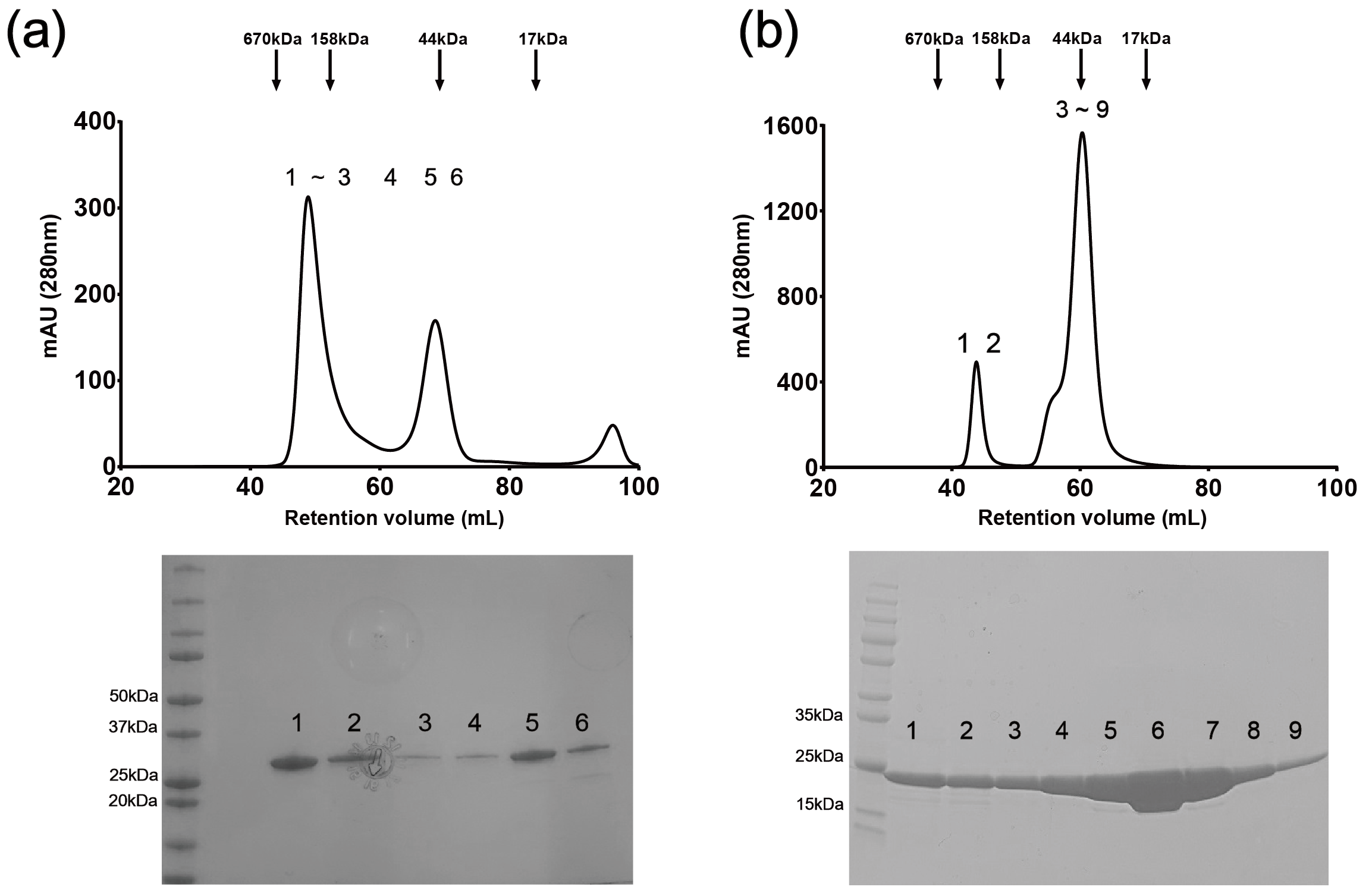


**Supplementary Figure 2. Oligmerization states of recombinant Rv0187 with the N-terminal or C-terminal hexa-histidine tag.** Elution profile of purified recombinant Rv0187 fused with the N-terminal (A) and C-terminal His6-tag (B) from a size exclusion column, where the predicted molecular weight of a monomer is 26.1 and 23.8 kDa respectively. Markers indicate the elution volume by a standard protein. Fractions from are displayed on SDS-PAGE gel stained with Coomassie-blue in each bottom panel.


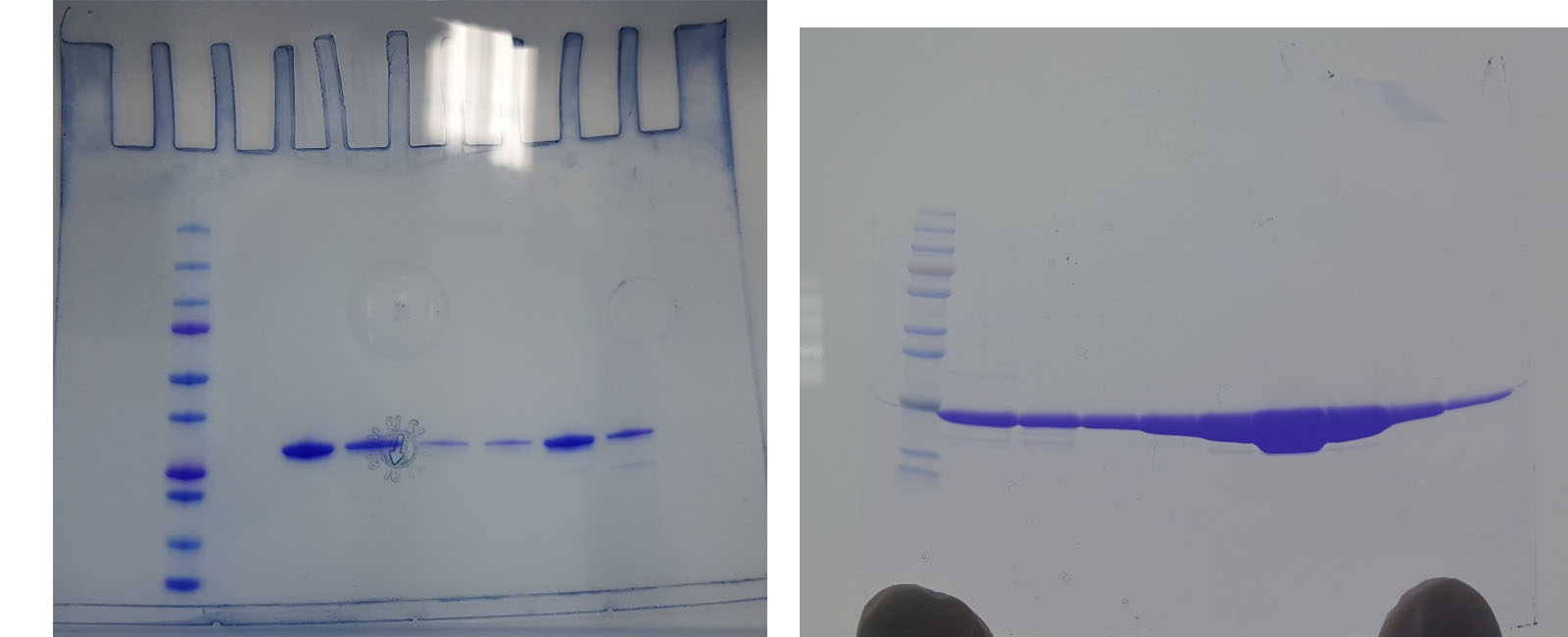


Full-sized picture of SDS gels used in Supplementary Figure 1.

**Supplementary Figure 3. Hydrophobic dimerization interface of Rv0187.** (a) One subunit of homodimer is presented with solvent accessible surface whereas the other in ribbon to facilitate the viewing of the dimeric interface. Electrostatic potential calculated from PDB2PQR server is mapped on the surface model of a monomer from ligand-free structure of Rv0187, where blue and red colors represent positive and red charge, respectively^2,3^. (b) A homodimer of Rv0187 is shown in backbone trace of alpha carbon atoms with amino acids participating in hydrogen bonding/ionic interactions at the dimer interface highlighted in sticks.


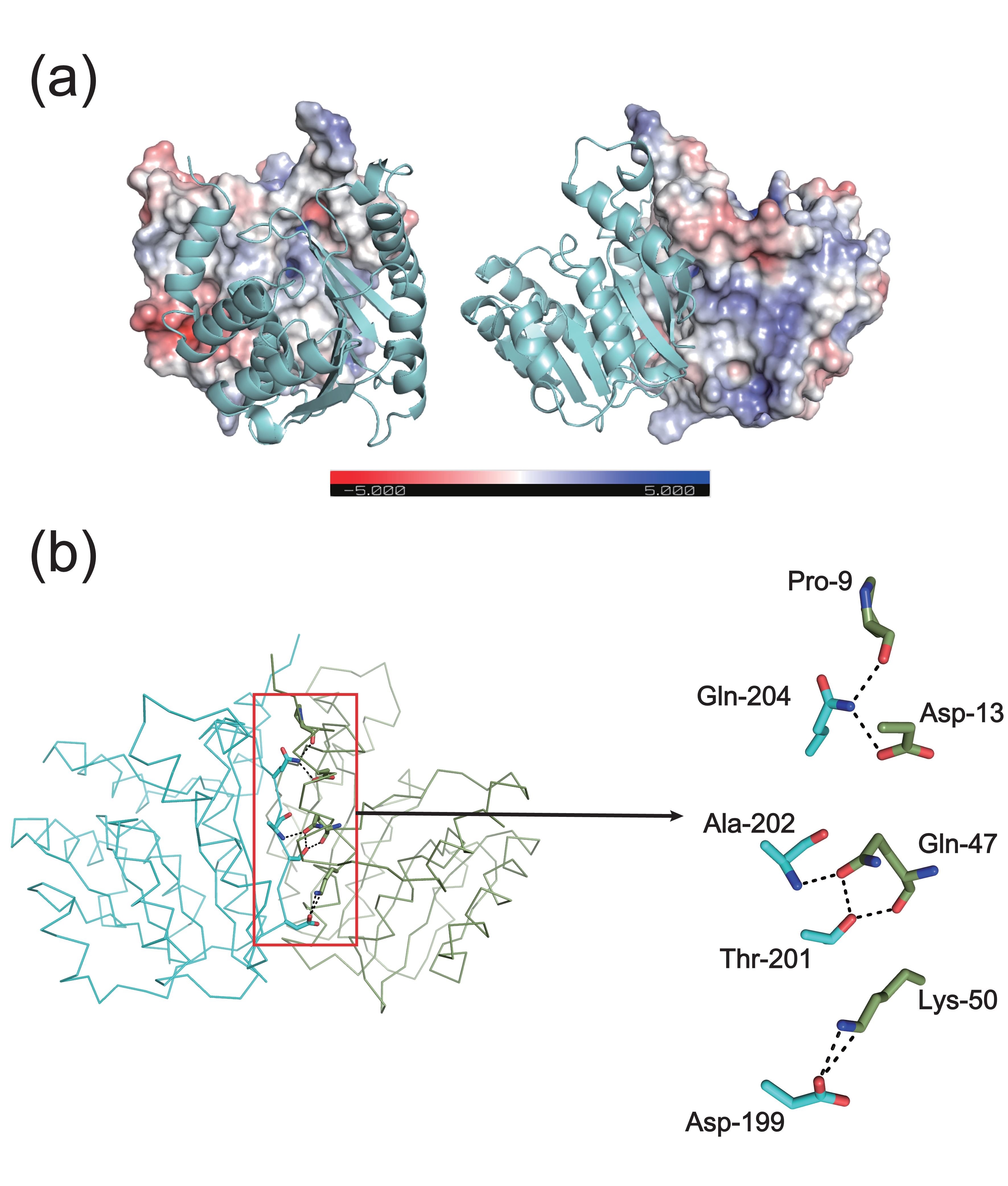


**
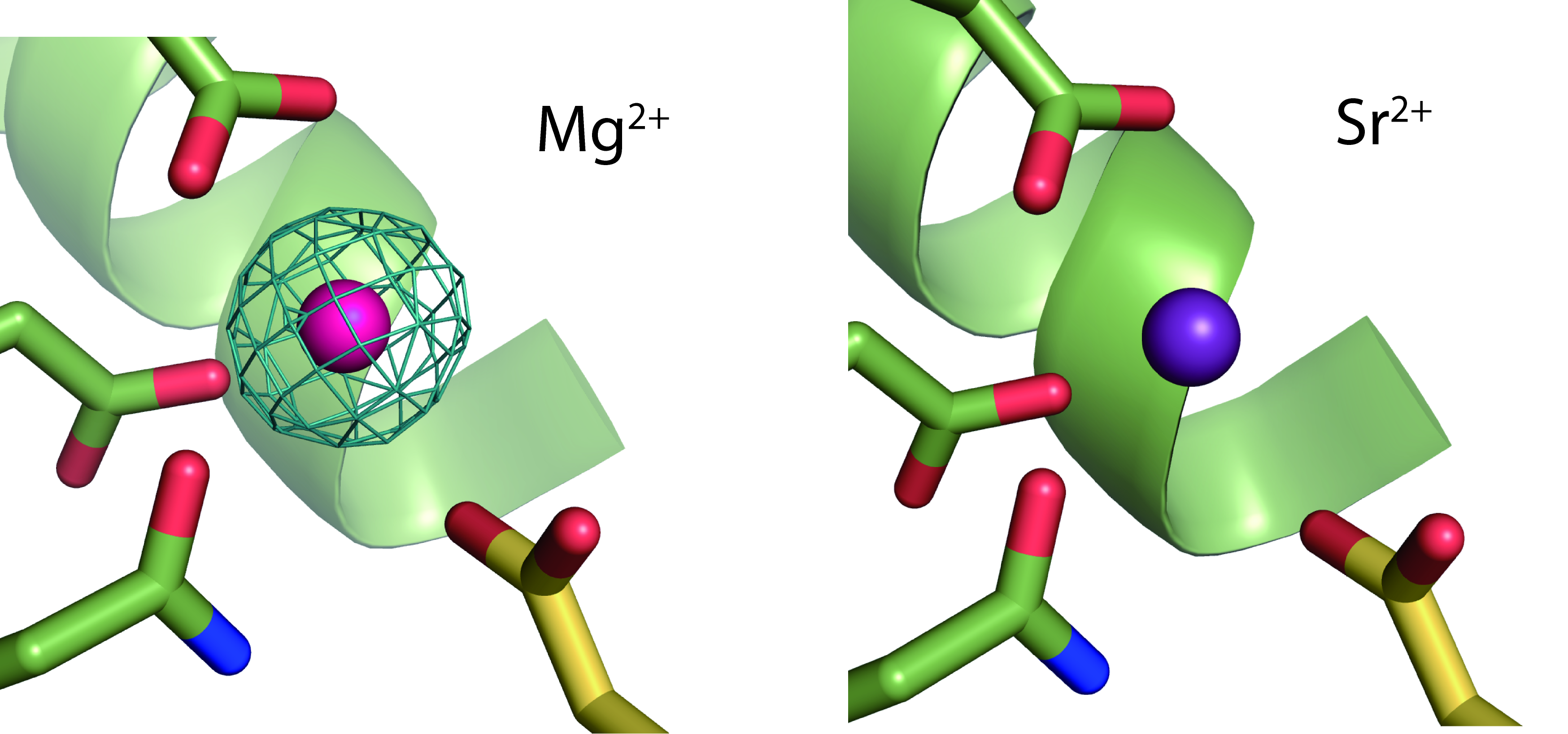
**

**Supplementary Figure 4. Omit electron density map around Mg^2+^or Sr^2+^.** Difference Fourier map (Fo - Fc) was calculated with magnesium (left) and strontium ion (right) in a model of cofactor-bound structure and shown as a green mesh contoured at 5.0 sigma.


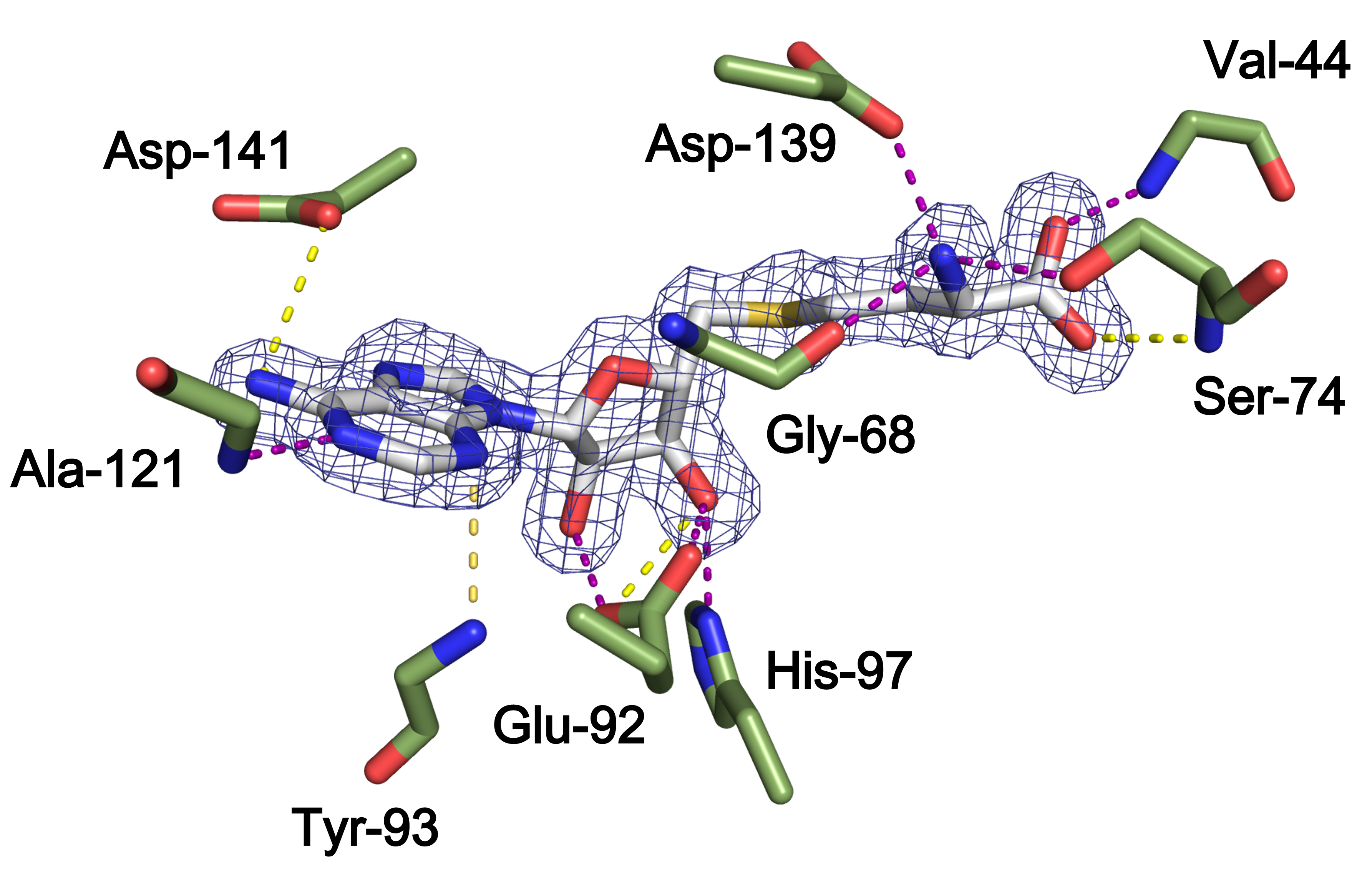


**Supplementary Figure 5. Difference Fourier map around SAH.**

2Fo-Fc difference Fourier map for SAH in a model of cofactor-bound structure is shown as a blue mesh contoured at 1.0 sigma with the interacting residues. The interactions found in all chains are shown in magenta, and the variable interactions in yellow.


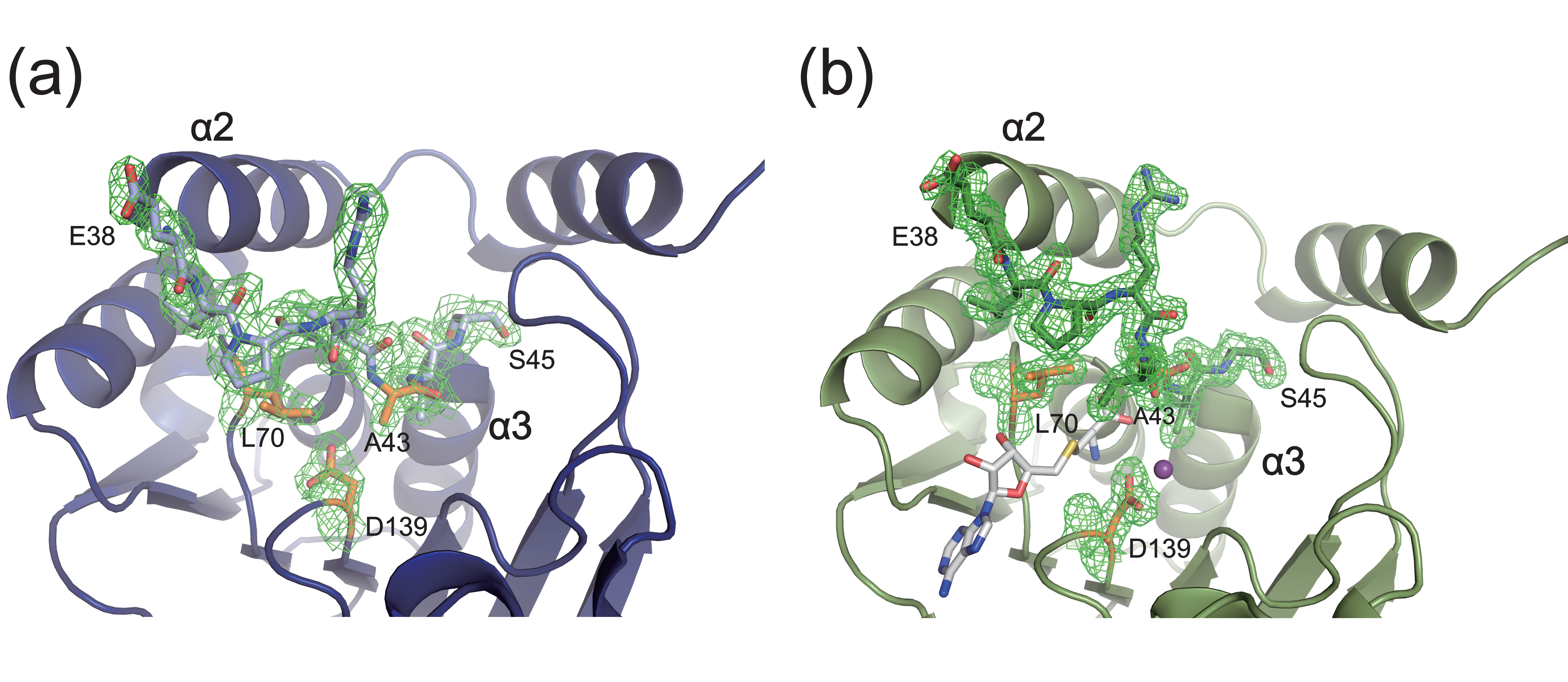


**Supplementary Figure 6. Difference Fourier map on the α2-α3 loop shown in Figure 4.**

The 2Fo-Fc electron-density maps over the amino acid residues 43 through 45 which defines the α2- α3 loop in the ligand-free (A) and cofactor-bound structures (B) are shown as a green mesh contoured at 1.0 sigma. Additionally, residues which would overlap with a strontium ion (Asp-139) and SAH (Ala-43 and Leu-70) in the overlaid structures are highlighted in orange.


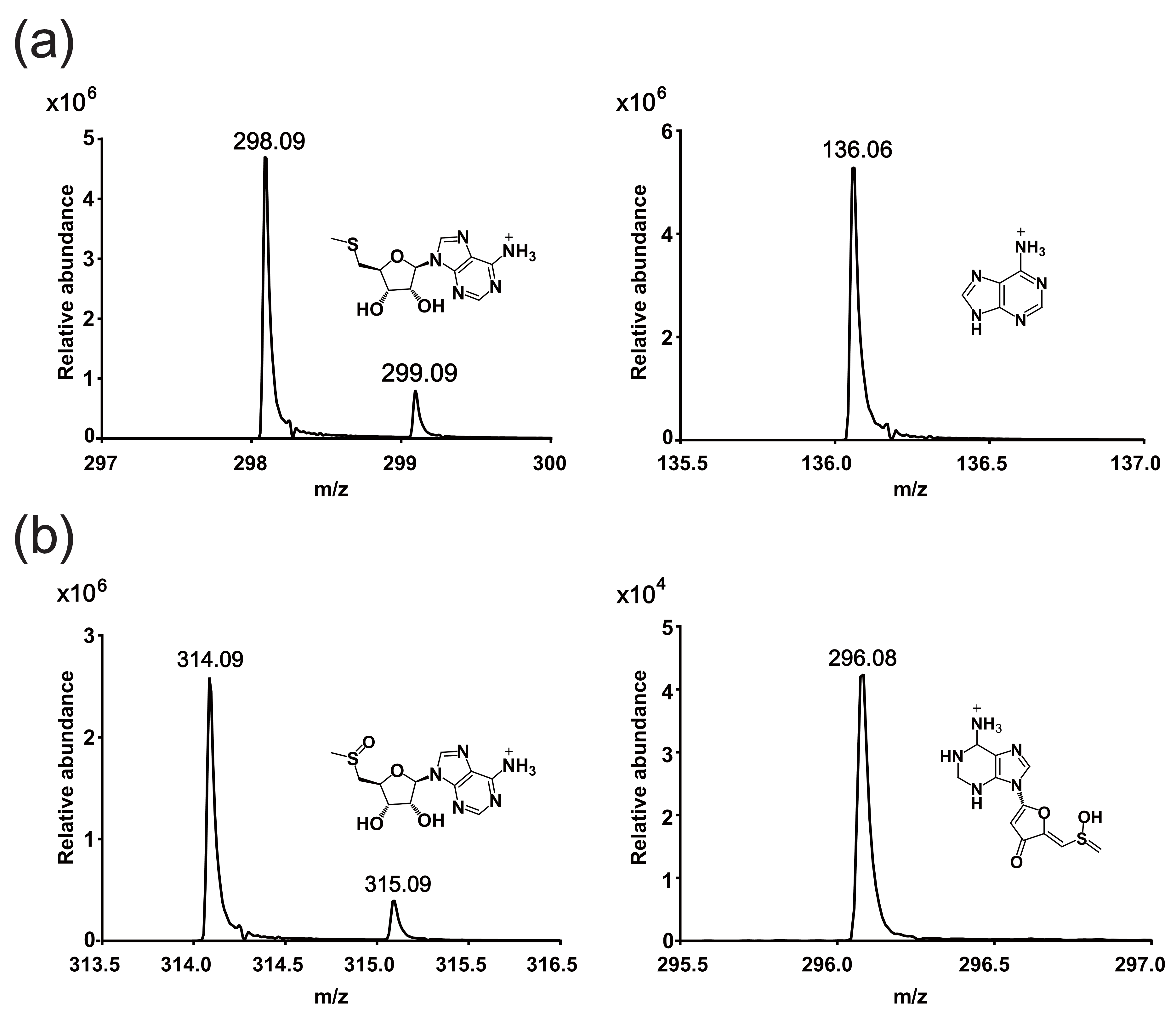


**Supplementary Figure 7. Analyses of ligands co-purified with recombinant Rv0187.**

Following the extraction of protein-bound ligands with 95% MeOH, the sample was analyzed by mass spectrometry where two major peaks were identified (left panels), and subsequent MS/MS fragment analysis were performed (right panels): A) **methylthioadenosine** ([M+H] = 298.09, calculated for C_11_H_16_N_5_O_3_S) **and B) MTA-SO** ([M+H] = 314.09, calculated for C_11_H_16_N_5_O_4_S).

**
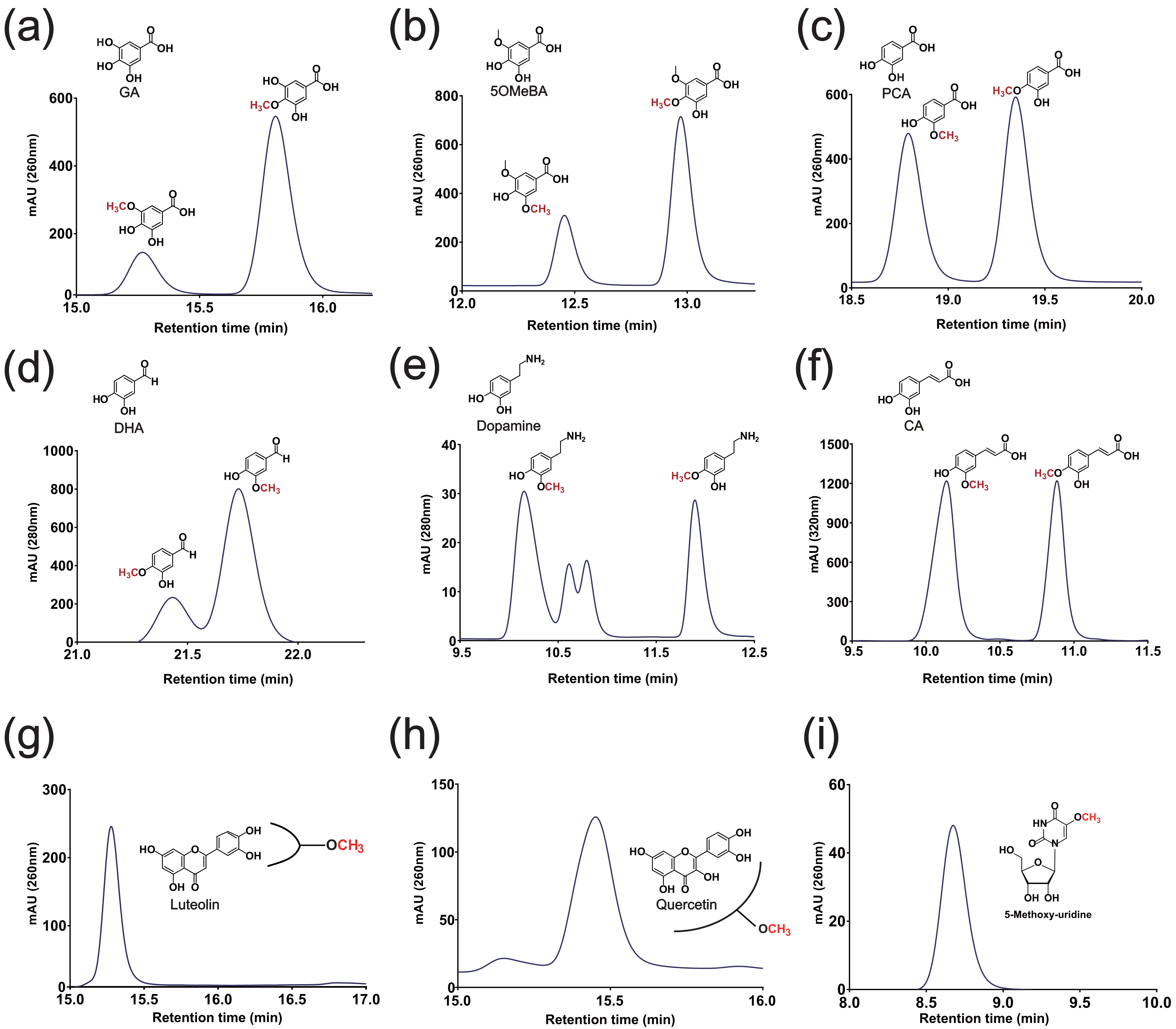
**

**Supplementary Figure 8. HPLC analyses of substrate specificity of Rv0187.**

HPLC traces of methylation products of a various catechol-like compounds from Rv0187-dependent *in vitro* assays at 37℃. Tested substrates are (A) gallic acid (GA), (B) 3,4-dihydroxy-5-methoxy-benzoic acid (5OMeBA), (C) protocatechuic acid (PCA), (D) 3,4-dihydroxy-benzaldehyde (DHA), (E) dopamine, (F) caffeic acid (CA), (G) luteolin, (H) quercetin, and (I) 5-hydroxyuridine. Assignment of each isomer was enabled by spiking with a standard compound except for (G) and (H).


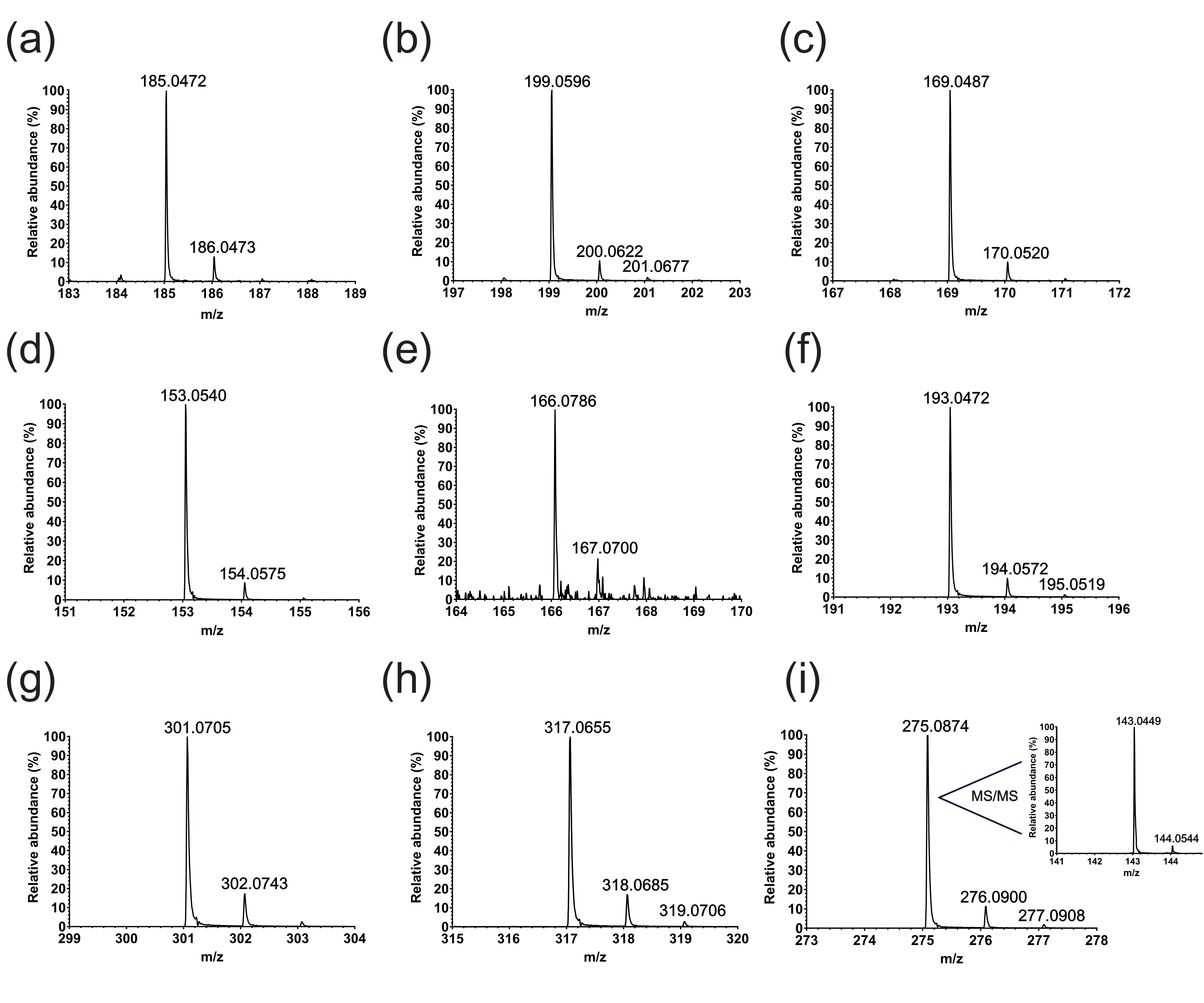


**Supplementary Figure 9. LC-MS analysis of Rv0187-dependent methyltransfer assays.**

A total of nine compounds with a catechol moiety were tested and the SAM-dependent methyltransfer activity was confirmed by LC-MS : (a) gallic acid (GA), (b) 3,4-dihydroxy-5-methoxy-benzoic acid (5OMeBA), (C) protocatechuic acid (PCA), (D) 3,4-dihydroxy-benzaldehyde (DHA), (e) dopamine, (f) caffeic acid (CA), (g) luteolin, (h) quercetin, (i) 5-hydroxyuridine. Following mass-to-charge ratios (m/z) were detected in positive ion mode: mono-methylated GA, m/z = 185.0472 (theoretical m/z = 185.0450, calculated for C_8_H_9_O_5_), mono-methylated 5OMeBA, m/z = 199.0596 (theoretical m/z = 199.0607, calculated for C_9_H_11_O_5_), mono-methylated PCA, m/z = 169.0487 (theoretical m/z = 169.0501, calculated for C_8_H_9_O_4_), mono-methylated DHA, m/z = 153.0540 (theoretical m/z =153.0552, calculated for C_8_H_9_O_3_), mono-methylated quercetin, m/z = 317.0655 (theoretical m/z = 317.0661, calculated for C_16_H_13_O_7_), mono-methylated luteolin, m/z = 301.0705 (theoretical m/z =301.0712, calculated for C_16_H_13_O_6_), and 5-methoxyuridine, m/z = 275.0874 (theoretical m/z = 275.0879, calculated for C_10_H_15_N_2_O_7_). LC-MS/MS analysis of the methylation of 5-hydroxy-uridine is displayed in the inset of (I). In negative mode, compounds with m/z of 166.0786 and 193.0472 were detected, consistent with mono-methylated dopamine (theoretical m/z = 166.068, calculated for C_9_H_12_NO_2_) and mono-methylated CA (theoretical m/z = 193.0501, calculated for C_10_H_9_O_4_), respectively.


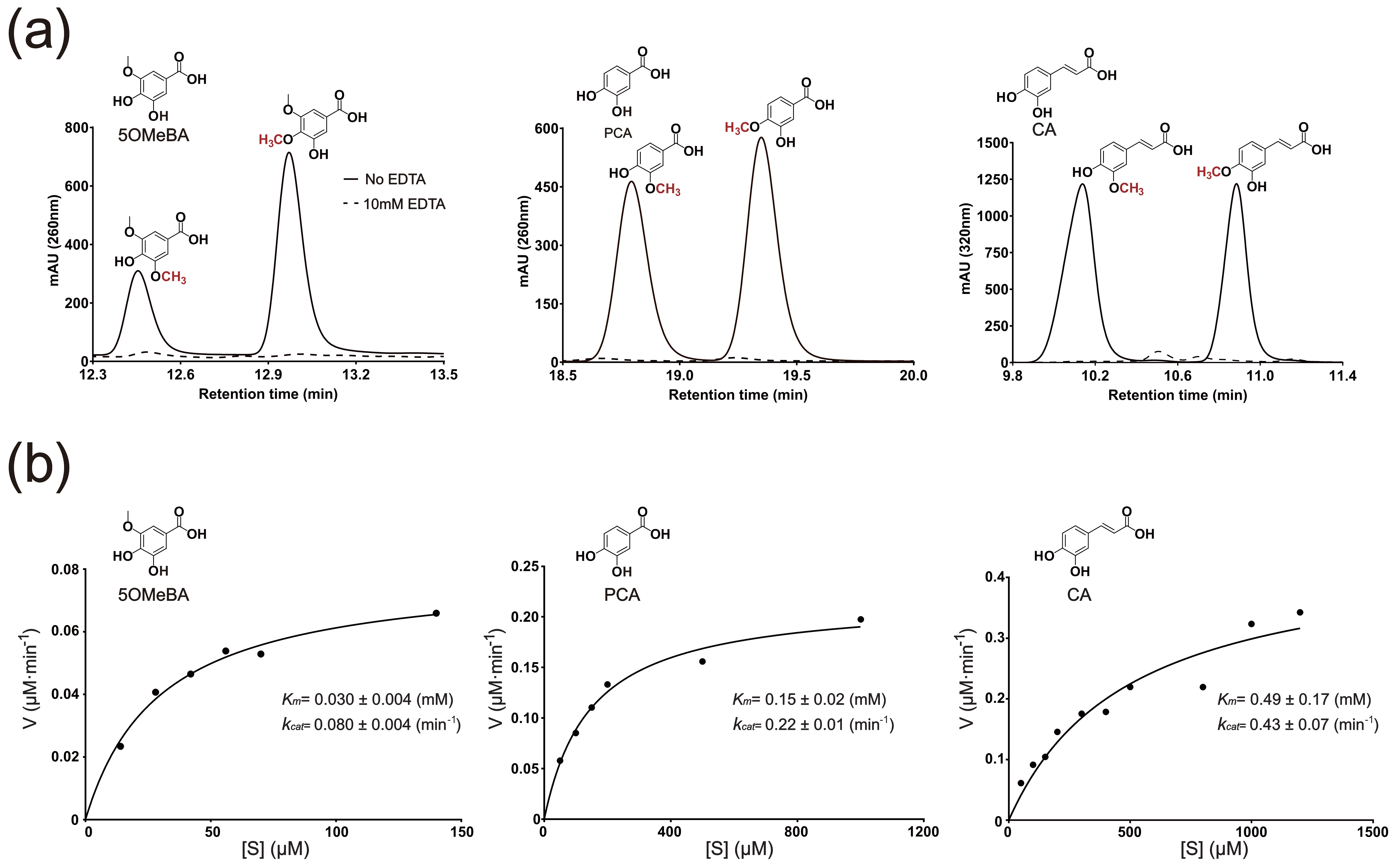


**Supplementary Figure 10. Michaelis-Menten kinetics of Rv0187.** (a) Metal-dependency of Rv0187 activity was tested and shown are the HPLC traces of assay mixtures of 5OMeBA, PCA or CA as a substrate. The reaction mixture is composed of 25 mM Tris-HCl (pH 7.5), 100 mM NaCl, 3 mM MgCl_2_, 1 mM of SAM, and 1mM of each substrate. The reaction was initiated by mixing with 10 μM Rv0187. Either 10mM EDTA (dotted line) or water (solid line) was immediately added to the assay mixture, which was then incubated for 16h before injection into HPLC. (b) Michaelis-Menten plots for each compound shown in (a) along with derived parameters.

**
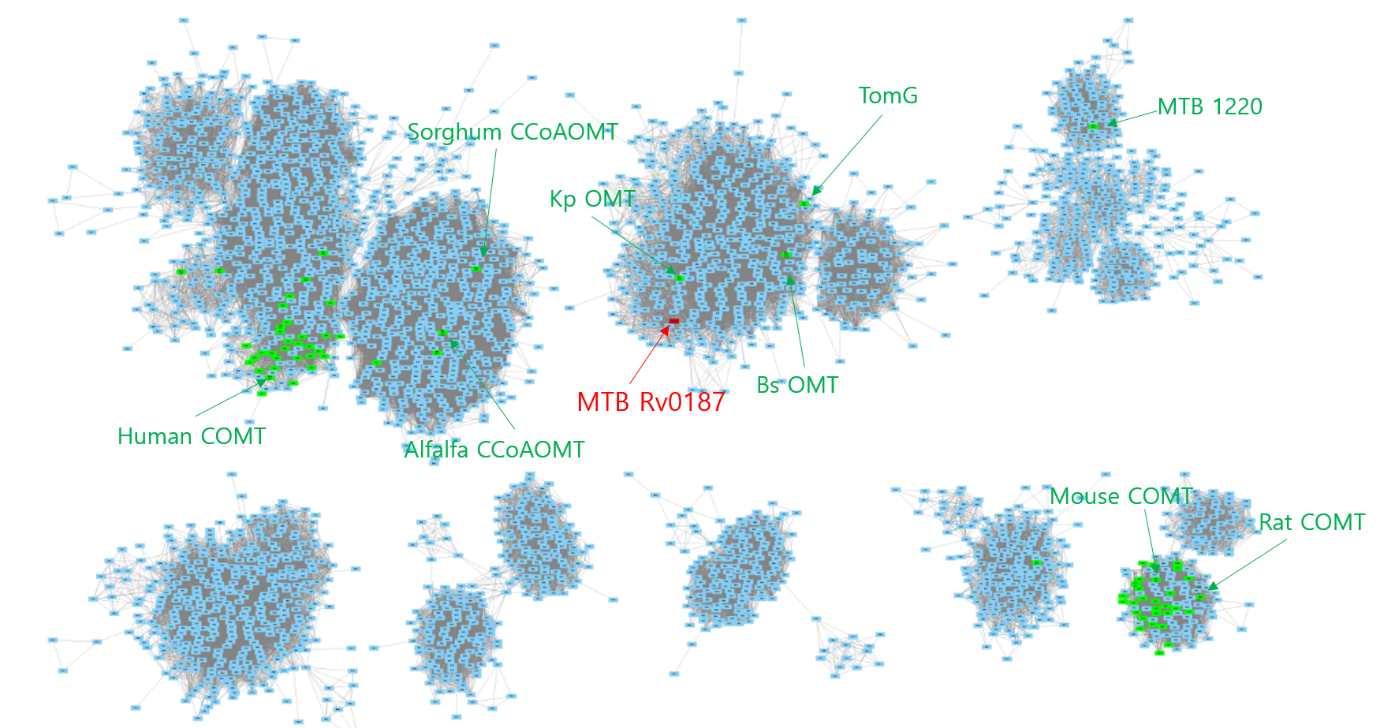
**

**Supplementary Figure 11. Sequence similarity network (SSN) for Pfam families PF01596.** Eight major clusters are shown for clarity, where nodes are represented in blue rectangles and edges are in grey lines. Nodes containing crystal structures are colored in green and indicated by arrows, and red node denotes Rv0187. SSN of PF01596 (O-methyltransferase family) was generated with Option B of the EFI-Enzyme Similarity Tool (EFI-EST) at an alignment score of 60, which uses one Pfam family as input^4^. Eight Network was visualized as 60% representative node networks. A total of 21,712 sequences were aligned using EFI-Enzyme Similarity Tool, which resulted in 6,332 nodes and 144,832 edges. The tool-generated data set was analyzed under an organic layout using the Cytoscape software.

**Supplementary Table1. Thermodynamic parameters of the binding interaction between Rv0187 and SAM determined by ITC**

|  | *K*_d_  (μM) | ΔG°  (kcal∙mol^−1^) | ΔH°  (kcal∙mol^−1^) | ΔS°  (cal/mol∙K) |
| --- | --- | --- | --- | --- |
| no metal | 9.3 ± 0.7 | − 6.82 ± 0.04 | − 6.31 ± 1.13 | 1.68 |
| +10mM MgCl_2_ | 12.5 ± 2.2 | − 6.71 ± 0.11 | − 6.51 ± 0.51 | 0.68 |
| +10mM SrCl_2_ | 12.5 ± 1.4 | − 6.69 ± 0.06 | − 7.45 ± 0.80 | −2.55 |

Each value and error represent averages and standard deviations from three independent experiments, respectively.

**Supplementary Table 2. Regioisomeric excess (*r.e*.) of Rv0187-dependent methylation**

|  | 25℃ | 37℃ | K142A (37℃) |
| --- | --- | --- | --- |
| GA | -61.6 (%) | -64.2 | 2.70 |
| 5OMeBA | -27.0 | -36.8 | -0.03 |
| PCA | -16.6 | -10.7 | 28.7 |
| DHA | 45.7 | 52.3 | 65.4 |
| Dopamine | 5.97 | 19.2 | 46.2 |
| CA | 10.1 | 11.5 | 29.8 |

+ and - values mean meta and para preference respectively.

**Supplementary Table 3. Steady-state kinetics parameters for Rv0187**

| substrate | *K_M_* (mM) | *k_cat_* (min^-1^) | *k_cat_*/*K_m_* (min^-1^·M^-1^) |
| --- | --- | --- | --- |
| 5OMeBA | 0.030 ± 0.004 | 0.080 ± 0.004 | 2.7 ×10^3^ |
| PCA | 0.15 ± 0.02 | 0.22 ± 0.01 | 1.47 ×10^3^ |
| CA | 0.49 ± 0.17 | 0.43 ± 0.07 | 0.88 ×10^3^ |

**Reference**

1. Robert, X. & Gouet, P. Deciphering key features in protein structures with the new ENDscript server. **42**, 320–324 (2014).
2. Baker, N. A., Sept, D., Joseph, S., Holst, M. J. & McCammon, J. A. Electrostatics of nanosystems: Application to microtubules and the ribosome. *Proc. Natl. Acad. Sci.* **98**, 10037–10041 (2001).
3. Dolinsky, T. J., Nielsen, J. E., Mccammon, J. A. & Baker, N. A. PDB2PQR : an automated pipeline for the setup of Poisson – Boltzmann electrostatics calculations. **32**, 665–667 (2004).
4. Gerlt, J. A. *et al.* Enzyme function initiative-enzyme similarity tool (EFI-EST): A web tool for generating protein sequence similarity networks. *Biochim. Biophys. Acta - Proteins Proteomics* **1854**, 1019–1037 (2015).
